# Impact of a conserved N-terminal proline-rich region of the α-subunit of CAAX-prenyltransferases on their enzyme properties

**DOI:** 10.1186/s12964-022-00929-w

**Published:** 2022-08-08

**Authors:** Anna Hagemann, Sandro Tasillo, Aykut Aydin, Miriam Caroline Alice Kehrenberg, Hagen Sjard Bachmann

**Affiliations:** Institute of Pharmacology and Toxicology, Centre of Biomedical Education and Science, School of Medicine, Faculty of Health, Witten/Herdecke University, Stockumer Strasse 10, 58453 Witten, Germany

**Keywords:** Human Farnesyltransferase, Proline-rich region, Interaction, Homodimer, Heterodimer, Prenylation

## Abstract

**Background:**

The CAAX-prenyltransferases farnesyltransferase (FTase) and geranylgeranyltransferase I (GGTase I) are heterodimers with a common α- (FTα) and unique β-subunits. Recently, α-subunits of species (e.g., human) that harbour an N-terminal proline-rich region (PRR) showed different dimerization behaviours than α-subunits without PRR (e.g., yeast). However, the specific function of the PRR has not been elucidated so far.

**Methods:**

To determine whether the PRR is a conserved motif throughout eukaryotes, we performed phylogenetics. Elucidating the impact of the PRR on enzyme properties, we cloned human as well as rat PRR deficient FTα, expressed them heterologously and compared protein–protein interaction by pull-down as well as crosslinking experiments. Substrate binding, enzyme activity and sensitivity towards common FTase inhibitors of full length and PRR-deletion α-subunits and their physiological partners was determined by continuous fluorescence assays.

**Results:**

The PRR is highly conserved in mammals, with an exception for marsupials harbouring a poly-alanine region instead. The PRR shows similarities to canonical SH3-binding domains and to profilin-binding domains. Independent of the PRR, the α-subunits were able to dimerize with the different physiological β-subunits in in vitro as well as in yeast two-hybrid experiments. FTase and GGTase I with truncated FTα were active. The K_M_ values for both substrates are in the single-digit µM range and show no significant differences between enzymes with full length and PRR deficient α-subunits within the species.

**Conclusions:**

Our data demonstrate that an N-terminal PRR of FTα is highly conserved in mammals. We could show that the activity and inhibitability is not influenced by the truncation of the N-terminal region. Nevertheless, this region shows common binding motifs for other proteins involved in cell-signalling, trafficking and phosphorylation, suggesting that this PRR might have other or additional functions in mammals. Our results provide new starting points due to the relevant but only partly understood role of FTα in eukaryotic FTase and GGTase I.

**Video Abstract**

**Supplementary Information:**

The online version contains supplementary material available at 10.1186/s12964-022-00929-w.

## Introduction

An important post-translational modification in eukaryotes is prenylation. It influences localization as well as activation of at least 200 different proteins [1]. Canonically, prenylation leads to membrane association and functionality of proteins in cell signalling pathways [2, 3]. In eukaryotes, four different enzymes, namely the farnesyltransferase (FTase) (EC 2.5.1.58) and three geranylgeranyltransferases (GGTase I (EC 2.5.1.59), II (EC 2.5.1.60) and III (no EC, yet)) are able to perform protein prenylation [4–6]. FTase and GGTase I catalyse the attachment of a farnesyl (15C) and geranylgeranyl (20C) moiety to the cysteine of the so called CAAX-box at the C-terminus of proteins [1, 7, 8]. Both enzymes are heterodimers with unique β-subunits but an identical α-subunit (FTα) [9, 10]. The catalytic centres as well as protein specificity of the enzymes are mediated by the β-subunits [11]. However, FTα is strictly required for enzymatic activity [12]. Both subunits form a very stable heterodimer thereby significantly increasing each other’s stability [13]. In 2015 it was shown that the truncated α-subunit of *Rattus norvegicus* (FTαΔ1-29) and full length FTα of *Saccharomyces cerevisiae* are capable of forming homodimers, assuming FTα might be involved in further intracellular signalling pathways [14]. However, our group was not able to prove homodimerization of native full-length human and rat FTα [15]. Notably, the artificially removed N-terminal part of rat FTα is a proline-rich region (PRR). Human FTα contains this PRR as well, but it is naturally absent in *Saccharomyces cerevisiae*. PRRs are known to be modulators of protein–protein interactions and due to this also for being necessary for activation [16–18]. Hence, the contrary dimerization behaviours might originate from the PRR. But PRR motifs are also known to be involved in signalling events, in the late stage of virus budding and in cell motility [16, 19, 20]. Therefore, the aim of this study was to further elucidate the occurrence and function of this PRR of FTα to get a hint whether there is an influence on the recruiting of its partners, the enzyme formation and activity. In particular we had the following questions: i) do we find the PRR in all species containing the FTase, ii) does the PRR have an influence on the binding behaviour of FTα to its physiological partners FTβ and GGT1β or to itself, iii) is there a change in enzyme activity of FTase and GGTase when the PRR is missing in human and rat FTα and iv) does the PRR of FTα fit to a known motif?

## Materials and methods

### Cloning and heterologous expression in *Escherichia coli*

*Escherichia coli* strains DH5α (Thermo Fisher Scientific, Waltham, MA, USA), BL21-CodonPlus(DE3)pLysS and Rosetta(DE3)pLysS (both Novagen, Darmstadt, Germany) for cloning and expression studies were cultured under standard conditions following the instructions of the manufacturers.

The cloning of the coding regions of full-length as well as truncated human and rat FTα (*FNTA*), FTβ (*FNTB*) and human GGT1β (*PGGT1B*) was performed as previously described [15]. The vectors used, antibiotic resistances, primer sets and restriction sites are summarized in Tables 1 and 2. Successful cloning was confirmed by sequencing (Seqlab, Göttingen, Germany). Cells were grown in LB-medium at 37 °C containing the appropriate antibiotics. Expression was induced by addition of 0.4 mM isopropyl-1-thio-β-D-galactopyranoside at an OD_600_ of ~ 0.6 (and 0.5 mM ZnSO_4_ for cells transformed with *FNTA* and *FNTB*, or *PGGT1B*, respectively). After induction cells were grown for 4 h at 34 °C [21], harvested by centrifugation (8000 × g, 4 °C, 20 min) and stored at − 80 °C.

**Table 1:** Primers used for cloning of the expression plasmids

No	Sequence 5’–3’	Restriction enzyme	Construct
1 for	*TTAAGG***GGATCC**GATGCAGCAGCAGCACAAG	*Bam*HI	pETDuet::h*FNTA* Δ1-31-His
1 rev	*GGCCGG***GTCGAC**TTATTGCTGTACATTTG	*Sal*I	
2 for	*TTCCGG***GAATTC**GATGCCAGCACAGCAGC	*Eco*RI	pETDuet::r*FNTA* Δ1-29-His
2 rev	*AAA***GTCGAC**CTATACACTCGCCGGTAT	*Sal*I	
3 for	*GGCCGG***AGATCT**AATGCAGCAGCAGCACAAG	*Bgl*II	pETDuet::h*FNTB*-His/ h*FNTA* Δ1-31
3 rev	*AAA***CTCGAG**TTATTGCTGTACATTTG	*Xho*I	
4 for	*AATTGG***AGATCT**ATGCCAGCACAGCAGC	*Bgl*II	pETDuet::r*FNTB*-His/ r*FNTA* Δ1-29
4 rev	*AAA***GGTACC**CTATACACTCGCCGGTAT	*Kpn*I	
5 for	*TTCCGG***GAATTC**GATGCAGCAGCAGCACAAG	*Eco*RI	pGEX-4T3::h*FNTA* Δ1-31-GST
5 rev	*GGCCGG***GTCGAC**TTATTGCTGTACATTTG	*Sal*I	
6 for	*TTCCGG***GAATTC**GATGCCAGCACAGCAGC	*Eco*RI	pGEX-4T3::r*FNTA* Δ1-29-GST
6 rev	*AAA***GTCGAC**CTATACACTCGCCGGTAT	*Sal*I	
7 for	*TTAAGG***GAATTC**GATGGCGGCCACTGAG	*Eco*RI	pETDuet::h*PGGT1B*-His
7 rev	*GGCCGG***GTCGAC**TCATGTGGAGATATGTAC	*Sal*I	

Itallic sequence indicates overhang; fat sequence indicates restriction site; for: forward primer; rev: reverse primer; pETDuet-1 and pGEX-4T3 are ampicillin-resistant; „h “ indicates *Homo sapiens* constructs; „r “ indicates *Rattus norvegicus* constructs; „Δ1-31/1-29” indicates location and number of truncated amino acids

**Table 2:** Primers used for cloning of the yeast two-hybrid constructs

No.	Sequence 5’–3’	Restriction enzyme	Construct
8 for	*TTCCGG***GAATTC**ATGCAGCAGCAGCACAAG	*Eco*RI	pGBKT7:: h*FNTA* Δ1-31
8 rev	*GGCCGG***GTCGAC**TTATTGCTGTACATTTG	*Sal*I	
9 for	*AATTGG***GAATTC**ATGCCAGCACAGCAGC	*Eco*RI	pGBKT7:: r*FNTA* Δ1-29
9 rev	*AAA***GGATCC**CTATACACTCGCCGGTAT	*Bam*HI	
10 for	*TTCCGG***GAATTC**GGATGCAGCAGCAGCACAAG	*Eco*RI	pACT2::h*FNTA* Δ1-31
10 rev	*GGCCGG***CTCGAG**TTATTGCTGTACATTTG	*Xho*I	
11 for	*AATTGG***CCATGG**GGATGCCAGCACAGCAGC	*Nco*I	pACT2::r*FNTA* Δ1-29
11 rev	*AAA***GAATTC**CTATACACTCGCCGGTAT	*Eco*RI	

Itallic sequence indicates overhang; fat sequence indicates restriction site; for: forward primer; rev: reverse primer; pACT2 are ampicillin-resistant; pGBKT7 is kanamycin-resistant; „h “ indicates *Homo sapiens* constructs; „r “ indicates *Rattus norvegicus* constructs; „Δ1-31/1-29” indicates location and number of truncated amino acids

### Purification of recombinant enzymes

To purify His-tagged prey proteins for GST pull-down and *in-vitro* crosslinking affinity chromatography with Ni-NTA was used as described previously [15]. In brief, *E. coli* cells were suspended (5 ml/1 g wet cell weight) in buffer A (50 mM Tris/HCl, pH 7.5, 300 mM KCl, 10 mM MgCl_2_, 10 µM ZnCl_2_, 20 mM imidazole, 5 mM DTT and protease inhibitor cocktail (Roche, Basel, Switzerland), for GST pull-down experiments) or buffer B (100 mM sodium phosphate buffer, 150 mM NaCl, 10 µM ZnCl_2_, for bis(sulfosuccinimidyl)suberate (BS^3^) crosslinking) and lysed by sonification (3 × 8 min) in an ice ethanol bath. Cell debris and unbroken cells were removed by centrifugation (21,100 × g, 45 min, 4 °C). The lysate was applied to a Ni–NTA IMAC (immobilized metal ion affinity chromatography) slurry (Qiagen, Hilden, Germany) equilibrated with buffer A or B, respectively, incubated in a rotating revolver at 4 °C for 90 min and applied to a column. After washing (3 × 10 ml buffer A or B by gravity flow) the column was incubated on ice for 5 min with 200 µl buffer AE (buffer A, 250 mM imidazole, for GST pull-down) or BE (buffer B, 250 mM imidazole, for BS^3^ crosslinking) and eluted by gravity flow.

For the purification of GST-tagged proteins as prey proteins for His pull-down experiments the MagneGST purification system (Promega, Mannheim, Germany) was used. *E. coli* cells (0.5 g wet cell weight) were dissolved in 500 µl MagneGST cell lysis reagent (adding 3–5 U RNase free DNase) and incubated on a shaking plate (4 °C, 30 min). Magnetic beads were equilibrated according to the manufacturer’s protocol with slight modifications (400 mM NaCl in binding/washing buffer). For binding, the beads were incubated with GST or FTα-GST lysate (4 °C, 30 min, shaking plate) and washed five times in binding/washing buffer. Proteins were eluted by incubation in 200 µl elution buffer (5 min, room temperature (RT), rotating plate) containing 50 mM glutathione.

### Pull-down assays

For GST pull-down assays the cells were treated as described above until the GST-proteins were bound to the beads and washed, then charged beads were resolved in 50 µl binding/washing buffer. Purified His-tagged FTα, 40 µl 10% BSA, 50 µl GST-charged beads and washing/binding-buffer (ad. 400 µl) were incubated (RT, 1 h, rotating plate) and washed five times. Elution was performed as described above.

### Immuno-blot and -detection

Protein fractions were analysed by SDS-PAGE and infra-red western blot (LiCor, Lincoln, NE, USA) with specific first antibodies (Anti-GST antibody [ERP4236] abcam (Cambridge, UK), anti-FTα antibody [ERP4704] abcam, anti-FTβ antibody [B-7], Santa Cruz (Heidelberg, Germany)) and infra-red second antibody (IRDye® 800CW goat anti-rabbit IgG, IRDye® 680RD goat anti-mouse IgG (Li-Cor)) or a coupled specific His-infra-red antibody (6x-His-tag antibody, 1:2000, DyLight 680, Thermo Fisher Scientific). After semi-dry blotting (30 min, 25 V) the nitrocellulose membrane was blocked (RT, 60 min, shaking) in blocking solution (Li-Cor), incubated with 1^st^ antibody (1:2000, blocking solution, 0.1% Tween20, RT, 60 min, shaking), washed (4 × 5 min TBS, 0.1% Tween20), and incubated with 2nd antibody (1:10,000, blocking solution, 0.1% Tween20, RT, 60 min, shaking in the dark). Finally, the membrane was washed (3 × 5 min, TBS, 0.1% Tween20, and 1 × 5 min TBS), dried in the dark and visualized on an Odyssey imager (Li-Cor).

### Yeast two-hybrid

In vivo protein–protein interaction was analysed using the Matchmaker system 3 Yeast two-hybrid system (Clontech, Mountain View, CA, USA). Coding regions of truncated FTα and FTβ were amplified by PCR and cloned using vectors and primer sets as summarized in Table 2. Successful cloning was confirmed by sequencing (Seqlab, Göttingen, Germany). Interaction of the proteins was detected by co-transformation of *S. cerevisiae* AH109 with pACT2 and pGBKT7 harbouring truncated *FNTA*, according to the manufacturer's instruction. In brief, competent AH 109 cells were incubated 5 min at 37 °C shaking with 5 µl pre-heated salmon sperm DNA (Sigma-Aldrich, Munich, Germany, 10 mg/ml, 10 min, 98 °C) and 1 μg of the respective plasmids. Afterwards 700 μl PEG-bicine solution (40% (w/v) PEG1000, 200 mM bicine, pH 8.35) was added to the cells (1 h, 30 °C). The cells were washed in NaCl-bicine (150 mM NaCl, 10 mM bicine), plated on selective SD-medium (-leucine, -tryptophan) and incubated (three days, 30 °C). As described previously, the interaction of truncated and/or full-length FTα and FTβ served as positive control. Yeast colonies were cultivated in liquid SD-medium (-leucine, -tryptophan), grown to an OD_600_ of ~ 1.5 and diluted to an OD_600_ of 0.1. From this dilution 5 µl were dropped on SD-plates (-leucine, -tryptophan, -histidine) supplemented with α-X-gal to check for α-galactosidase activity [22].

### In vitro* protein crosslinking*

The oligomerization-state of truncated human and rat FTα was analysed by crosslinking 8 µg of protein after affinity chromatography (as described above) with 50-fold BS^3^, according to the manufacturer’s protocol (Thermo Fisher Scientific). The reaction was stopped by the addition of 1 M Tris/HCl (pH 7.5, final concentration 50 mM) and the samples applied to a 12% SDS-PAGE, blotted on a nitrocellulose membrane, and analysed as described previously. As positive control purified truncated FTα and FTβ were used (rat and human). For negative control according to the manufacturer’s protocol, 2% β-mercaptoethanol was added to the reaction mixture to inhibit the crosslinking reaction.

### Native size determination with size exclusion chromatography

To determine the native size of truncated FTα of rat and human, size exclusion chromatography (SEC) was used (Superdex 200 HiLoad 16/600 columns (GE Healthcare Life Sciences, Freiburg, Germany)). Therefore, FTα was expressed and purified by IMAC as described above. The resulting elution fractions were dialyzed over night at 4 °C in anion exchange chromatography buffer (50 mM Tris/HCl pH 7.5, 5 mM MgCl_2,_ 3 mM DTT), applied to the UNO Q6 monolith ion exchange column (BioRad, Hercules, CA, USA) and eluted with an increasing NaCl gradient (from 0 to 1000 mM). The resulting peaks were analysed by SDS-PAGE, the FTα containing fractions concentrated by Vivaspin 2, (30 kDa, Sartorius, Stonehouse, UK), dialysed against SEC buffer (50 mM Tris/HCl pH 7.5, 5 mM MgCl_2_, 3 mM DTT, 300 mM NaCl), applied to a SEC column (HiLoad 600 superdex 200 pg column, GE healthcare) and eluted by isocratic flow. The resulting peaks were analysed by SDS-PAGE for purity. Different amounts of pure protein (2, 3 and 5 µg) were applied to a native PAGE (BioRad) and analysed by Coomassie staining and immuno-blot as described.

### Determination of K_M_ of FTase/GGTase with truncated FTα

For determination of Michaelis–Menten constants (K_M_-value) of Dansyl-GCVLS and Danysl-GCVLL, respectively, the concentration of the substrate was varied (GCVLS 0–10 µM / GCVLL 0.75 -12 µM) while FPP and GGPP were kept constant at a saturating substrate concentration of 10 µM. Accordingly, for K_M_-values of FPP and GGPP, concentration was varied (FPP 0–20.8 µM / GGPP 0–20 µM), while the concentration of Dansyl-GCVLS / Dansyl-GCVLL was constantly saturated at 8 µM.

The enzyme activity was analysed using a continuous fluorescence assay with IMAC-purified enzymes in black flat 96-well plates (Thermo Fisher Scientific) with a total volume of 250 µL [8]. Different concentrations of peptide substrate Dansyl-GCVLS / Dansyl-GCVLL were preincubated with 5 mM DTT in H_2_O (total volume 50 µL) for 30 min at room temperature. Preincubated Dansyl/DTT solution and 120 µL of enzyme solution (2 µg enzyme solution in H_2_O) were incubated in assay buffer (final buffer concentrations of 50 mM Tris-HCl pH 7.5, 10 µM ZnCl_2_ and 0.03% n-Dodecyl-ß-D maltoside) for 3 min at 30 °C. The reaction was started adding 30 µL FPP/GGPP solution in varying concentrations.

The increase in fluorescence was measured every 30 s over a period of 60 min (extinction: 340 nm, emission: 505 nm). All measurements were performed at 30 °C using a Tecan microplate reader (Tecan Infinite 2000 PRO mPlex, Tecan group Ltd, Switzerland).

Additionally, we performed first experiments analysing the inhibitability of the truncated enzymes. The enzyme was treated as described with the difference, that increasing concentrations of lonafarnib or tipifarnib (Selleckchem, Houston, TX, USA) were added prior to the measurement. The change in fluorescence is shown time dependently.

### Determination of apparent ***k***_***cat***_

Enzyme activity measured as increase in fluorescence units per second (R) was converted to substrate turnover (v) in µM/s using following equation [23]:

$$v = \frac{RP}{{\Delta F}} \left[ {{\upmu }M/s} \right]$$

P is the amount of product formed in the assay and ΔF is the difference in fluorescence intensity between the start and the end of the reaction when fluorescence intensity is constant for several minutes.

Apparent k_cat_ was calculated from a fit of the Michaelis Menten equation to the initial velocity vs. substrate concentration.

For FTase, k_cat_ calculation was performed with the substrate Dansyl-GCVLS and for GGTase with substrate Dansyl-GCVLL. Two µg protein was used per measurement. The calculation was performed with GraphPad prism 8.0.

k_cat_ is reported as apparent value, since the enzyme purity was estimated to be approximately 80%. All samples were purified the same way by Ni-NTA and the purity was comparable.

### In silico analysis and statistics

Blast analysis was performed using NCBI [24, 25] as well as ClustalO [26, 27]. Analysis of the consensus sequence was performed by JalView [28]. Statistical analysis was performed using GraphPad Prism version 7.00 for Windows (GraphPad Software, La Jolla, USA). The determined K_M_ and k_cat_ values (nonlinear curve-fitting) were analysed for significance using t-test with α = 0.05. The K_M_ values are presented as the mean with 95% confidence interval. Comparison of K_M_ values to known data was performed with the database platform BRENDA [29]. k_cat_ values are presented as mean ± standard error of the mean (SEM).

## Results

### Appearance of the proline-rich region in different phyla

**Fig. 1:**
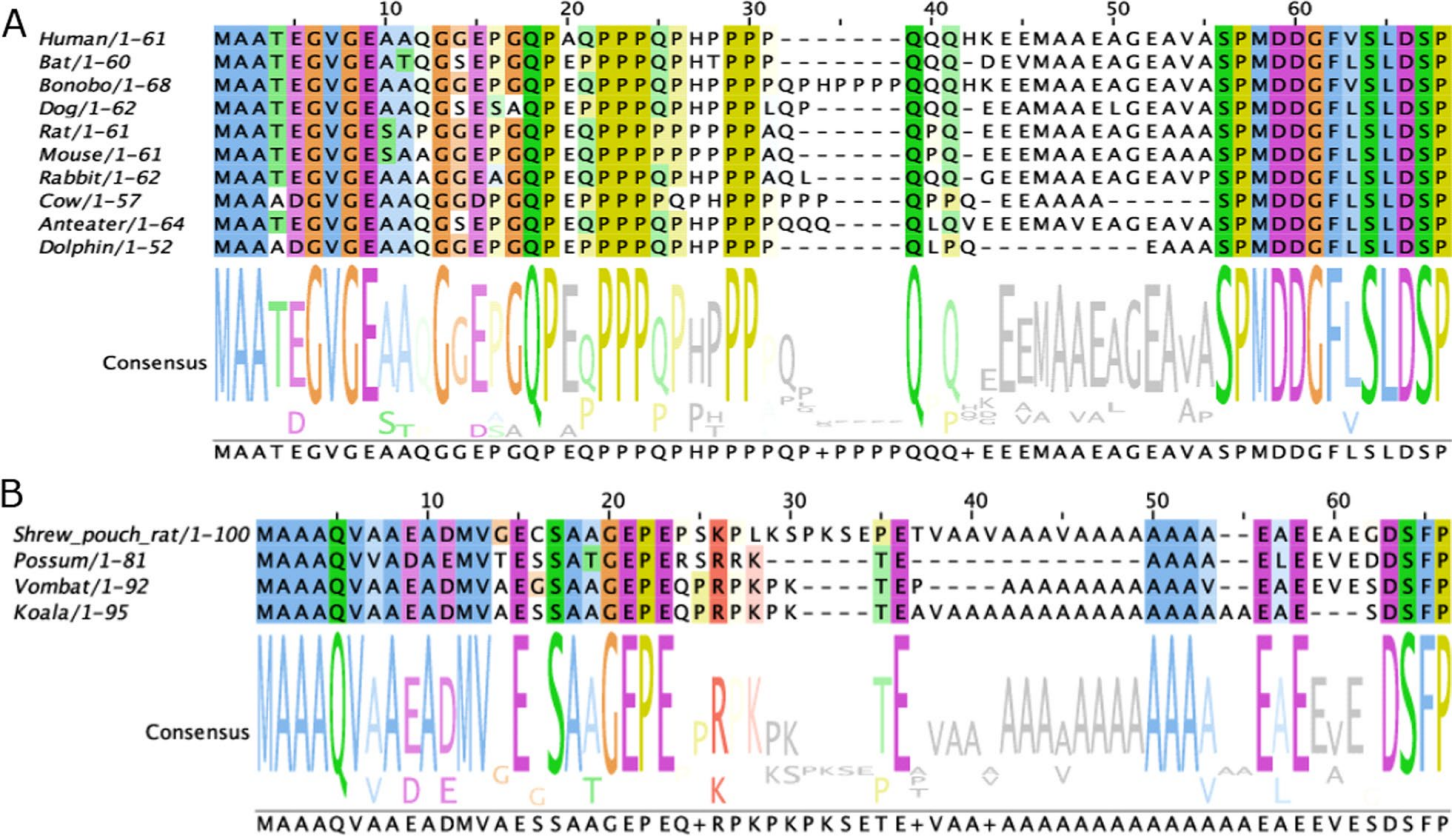
Alignment and consensus sequence of mammalian FTαs. **A** N-terminal part of representative mammalian FTα sequences (see Additional file 1: Fig. S4 for full set). **B** N-terminal part of representative marsupial FTα sequences (see Additional file 1: Fig. S4 for full set). Coloured letters indicate conservation > 30% according to clustalO. The consensus sequence is given below

The N-terminal part of human FTα comprises a PRR. To get a hint, how strong the conservation of the PRR in different phyla is, we performed multiple sequence alignments (Fig. 1 and Additional file 1: Fig. S1). Since neither homologues for *FNTA*, nor for *FNTB* can be found in prokaryotes, we analysed a broad range of eukaryotes. Lower creatures, like plants, yeasts and reptiles do not haves a PRR (Additional file 1: Fig. S1). This is also true for birds and fishes. Interestingly, mammals possess the PRR with exception for metatheria (marsupials) (Fig. 1A, B). Instead, marsupials harbour an alanine rich region, leading to the question, whether poly-alanine can fulfil the same role as prolines in this particular case (Fig. 1B). However, the amino acid sequence of vertebrates is highly conserved after amino acid 63 (reference NP_002018.1). Other higher eukaryotes share high sequence identity as well. Only plants and lower eukaryotes show more deviating sequences (Additional file 1: Fig. S1).

### Interaction of PRR deficient FTα with FTβ

**Fig. 2:**
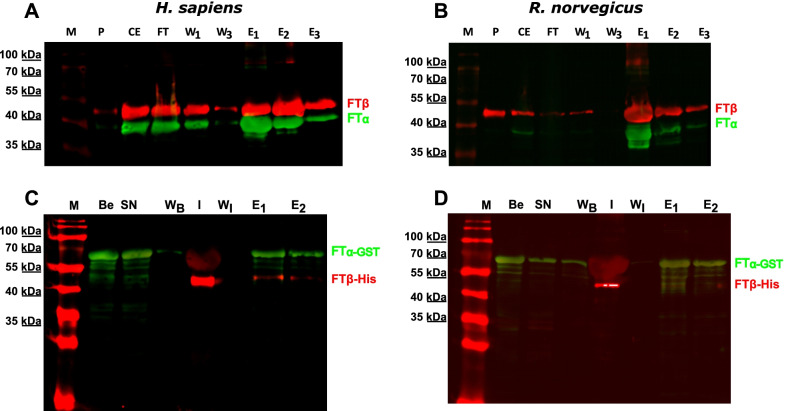
Immunoblots of co-expression and GST pull-downs. **A** and **B** (co-expression and IMAC), P (pellet), CE (crude extract), FT (flowthrough), wash 1 and 3 (W1, W3) and the 1st three elution fraction (E1, E2 and E3) are shown. The membranes were incubated with anti-His antibody (red) and anti-FNTA-antibody (green); **C** and **D** GST-pulldown of truncated human and rat FTα-GST with FTβ, fractions: beads (B), supernatant (SN), wash of the beads (W_B_), interaction (I), wash after interaction (W_I_) and elution 1 and 2 (E1, E2) The membranes were incubated with anti-His antibody (red) and anti-GST antibody (green). All samples were run on 12% SDS-PAGE and blotted by semi-dry blot

To elucidate whether the PRR has an impact on heterodimerization of FTα with FTβ, we performed heterologous coexpression of both subunits in *E. coli*, leading to a quite high protein yield since the full-length subunits are known to stabilize each other [13]. This could also be observed for the truncated variants of FTα (Fig. 2A, B). In the elution fraction of human and rat IMAC a bold band of FTβ (His-tagged) and co-eluted FTα appear, leading to the assumption that the truncated FTα subunits bind to FTβ.

Since there is an artificial tag at the N-terminus of FTβ, we performed the assay the other way around, as well. This time, the truncated FTα subunit has an N-terminal GST-tag. Purified His-tagged FTβ was applied to GST-FTα bound to magnetic beads (Fig. 2C, D). GST-FTα was able to capture His-FTβ indicating that there is a direct interaction of the two subunits.

**Fig. 3:**
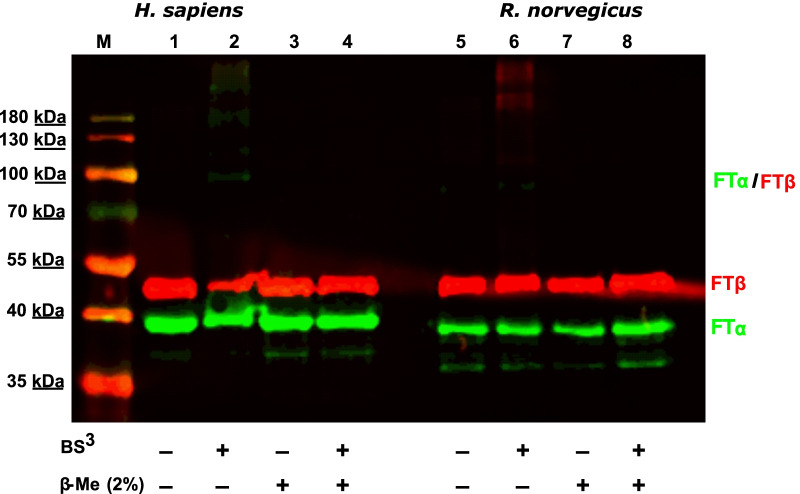
Immunoblots of FTα / FTβ BS^3^ crosslinking of human and rat. Affinity chromatography purified protein (FTα and FTβ (each 8 µg)) with 50-fold molar excess of BS^3^ with regard to the protein. As negative control 2% of the crosslinking inhibitor β-mercaptoethanol (β-Me) was added. All samples were applied to a 12% SDS-PAGE and membranes were incubated with anti-FNTB antibody (red) and anti-FNTA antibody (green)

To confirm the formation of a heterodimer BS^3^ crosslinking was performed with human and rat FTase subunits. This way the size of the resulting complex from FTβ and the truncated FTα subunit can be estimated (presumably 89 kDa). It is well known, that FTα appears around 5 kDa bigger on SDS-PAGE. Andres et al. proposed that this is due to the PRR [13]. On the immuno-blot a band of approximately 90 kDa can be observed after addition of the crosslinking reagent but not after addition of β-mercaptoethanol (Fig. 3). This finding supports the hypothesis that the PRR is responsible for the unusual migration of FTα.

### Dimerization behaviour of PRR deficient human and rat FTα

**Fig. 4:**
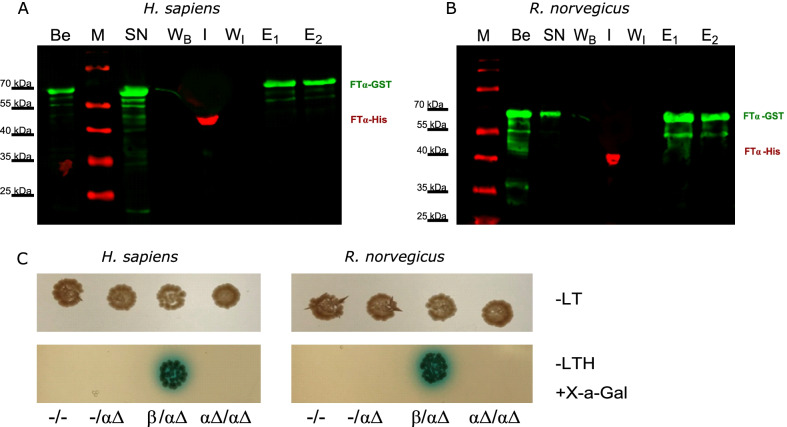
Self-binding of truncated human and rat FTα. Immunoblots of GST pull-downs of truncated **A** human and **B** rat FTα-GST with FTα. Applied fractions: beads (B), supernatant (SN), wash of the beads (W_B_), interaction (I), wash after interaction (W_I_) and elution 1 and 2 (E1, E2). All samples were run on 12% SDS-PAGE and blotted by semi-dry blot. The membranes were incubated with anti-His antibody (red, against tagged-FTα) and anti-GST antibody (green). C: Yeast two-hybrid analysis of truncated FTα interaction. Yeast strain AH109 was co-transformed with pACT2 and pGBKT7 constructs and dropped on different selective SD-agar plates. As positive control pACT2::*FNTB* and pGBKT7::*FNTA* were used. Upper panel: -leucine, -tryptophan (-LT). Lower panel: -leucine, -tryptophan, -histidine (-LTH), + X-α-gal

The self-dimerization of truncated human and rat FTα was assayed by GST pulldown experiments. Therefore, purified His-hFTαΔ1-31 or His-rFTαΔ1-29 and the respective GST-FTα bound to magnetic beads (Fig. 4A, B) were incubated together. The variants of GST-FTα (human or rat truncated FTα) were not able to capture their respective truncated His-FTα indicating that there is no direct interaction of the truncated alpha-subunits.

As an in vivo approach, we used the yeast two-hybrid system to analyse putative interactions. Formation of the truncated FTα − FTβ heterodimer (human as well as rat) served as positive control, whereas the empty vectors and accordingly one empty vector and one harbouring the respective truncated FTα construct, served as negative and false positive controls (Fig. 4C). Blue colonies on selective α-X-gal agar plates, indicating an interaction of the two protein partners, could only be observed when the truncated α- and β-subunit were present.

### Oligomerization behaviour of PRR deficient human and rat FTα

Human as well as rat His-FTα (Δ1-31 and Δ1-29, respectively) was analysed by size-exclusion chromatography. Analysing the peaks by SDS-PAGE confirmed a peak comprising truncated FTα. The analysis of the fractions for human and rat were analysed by a native PAGE (Additional file 1: Fig.S2) showing bands at the height of approx. 130 and 200 kDa. Since we already could detect this phenomenon for the full-length FTαs, we also had a closer look at the oligomerization of the truncated proteins.

**Fig. 5:**
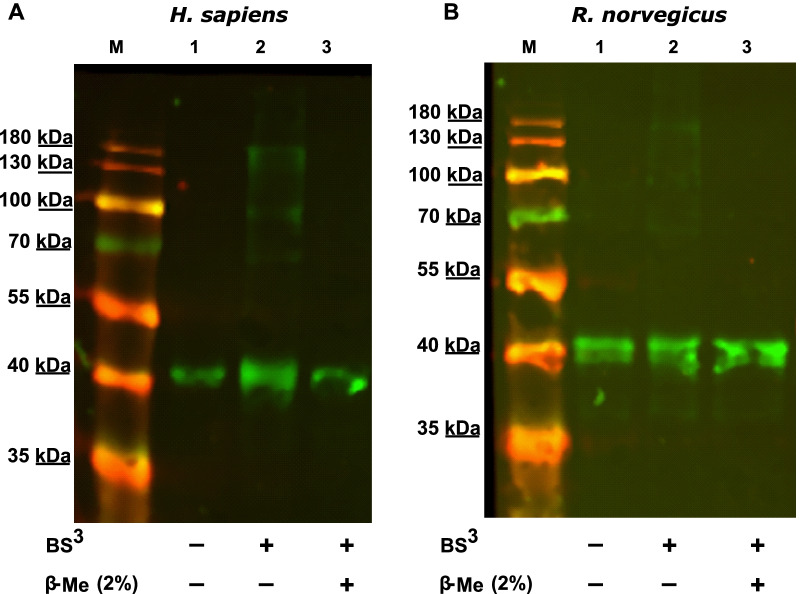
Immunoblots of truncated FTα BS^3^ crosslinking of human and rat. Affinity chromatography purified protein (8 µg) of **A** human and **B** rat FTα were incubated with 50-fold molar excess of BS^3^ with regard to the protein. As negative control 2% of the crosslinking inhibitor β-mercaptoethanol (β-Me) was added. All samples were applied to a 12% SDS-PAGE and membranes were incubated with anti-FNTB antibody (red) and anti-FNTA antibody (green)

In our previous study, we could show the formation of higher oligomers when incubating IMAC-purified FTα with the crosslinking agent BS^3^ [15]. The same experiment was performed with the truncated versions of human and rat FTα. The detected bands appear at sizes that do not match the size of a dimer. There is an even smaller band lower than 70 kDa and higher bands at 100 and 180 kDa that seem to result from unspecific binding. Interestingly, there is no specific band at the calculated size of the homodimer (~ 76 kDa) (Fig. 5).

### Determination of FTase activity dependent on FTα PRR

**Fig. 6:**
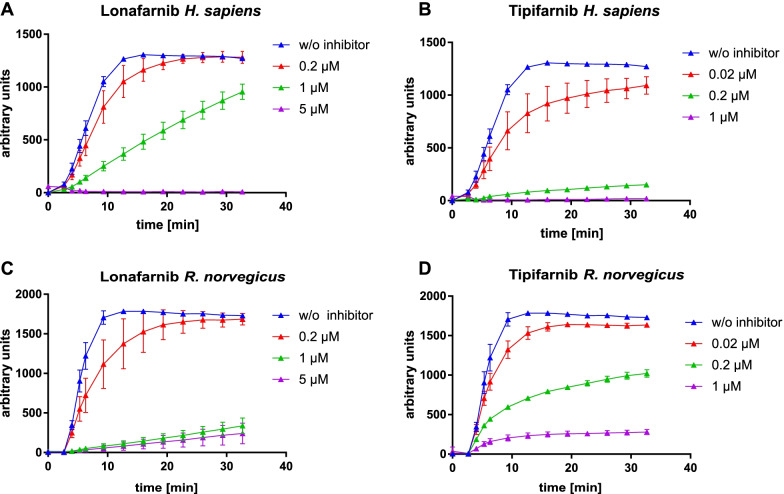
Inhibitor testing of human and rat FTα. Inhibition of human (**A** and **B**) and rat (**C** and **D**) FTase with truncated FTα was tested with lonafarnib (**A**, **C**) and tipifarnib (**B**, **D**). The purified enzyme was incubated with its substrates and different concentrations of lonafarnib and tipifarnib, respectively. The change in fluorescence was monitored over time every 30 s

To elucidate whether human and rat FTase can fulfil their physiological role without the N-terminal PRR of FTα, we performed a continuous fluorescence assay, measuring the change of fluorescence as a result of farnesylation of the substrate dansyl-GCVLS [8, 30]. All FTase variants investigated were enzymatically active. Comparison of the data of FTases containing full-length FTα with the truncated versions showed only minor differences (Tables 3 and 4). Since FTase has two substrates, FPP and a peptide substrate (here dansyl-GCVLS), we determined the K_M_-values for both. The K_M_-values for FPP of full-length human and rat FTase were quite similar (3.34 µM and 2.66 µM). Comparing these values with those of truncated human and rat FTα (4.00 μM and 5.27 μM) showed no significant differences (FPP K_M_ full-length vs. truncated: *p*_human_ 0.518; *p*_rat_ 0.179). This is also true for the K_M_-values for dansyl-GCVLS of full-length (2.22 µM and 2.32 µM) and truncated (1.56 µM and 2.36 µM) FTα (dansyl-GCVLS K_M_ full-length vs. truncated: *p*_human_ 0.313; *p*_rat_ 0.855). Furthermore, the analysis of the apparent k_cat_ values of full-length and truncated enzymes revealed no significant differences. In summary, presence or absence of the PRR does not influence the activity of human and rat FTase. Additionally, we could demonstrate that the most prominent FTase inhibitors, tipifarnib and lonafarnib, could still inhibit the FTase in a concentration dependant manner (Fig. 6), so the PRR does not influence enzyme inhibition. Lonafarnib and tipifarnib are competitive inhibitors against the peptide-substrate. Therefore, we assume, that the deletion of the PRR does not affect the peptide binding site.

**Table 3:** Michaelis–Menten constants in presence of FPP and Dansyl-GCVLS (farnesylation) or GGPP and Dansyl-GCVLL (geranylgeranylation)

FTα with FTβ	K_Μ_ FPP (95%CI) [µM]	K_Μ_ D-GCVLS (95%CI) [µM]
Human full-length	3.34 (2.73–4.10)	2.22 (1.53–3.24)
Human Δ1-31	4.00 (2.48–6.33)	1.56 (0.88–2.63)
Rat full-length	2.66 (2.01–3.52)	2.32 (1.46–3.73)
Rat Δ1-29	5.27 (3.79–7.32)	2.36 (1.48–3.71)

FTα with GGT1β	K_Μ_ GGPP (95%CI) [µM]	K_Μ_ D-GCVLL (95%CI) [µM]
Human full-length	1.13 (0.65–1.80)	4.50 (3.24–6.19)
Human Δ1-31	1.57 (0.94–2.56)	2.36 (1.36–4.06)

Data are presented as mean and 95% confidence interval (95%CI) is given. The measurements are performed at least three times

**Table 4:** Apparent k_cat_ for FTase and GGTase for their responding peptide substrate (FTase: Dansyl-GCVLS, GGTase: Dansyl-GCVLL)

	k_cat_	(SEM)	*p*-value
*FTα with FTβ*			
Human full-length	0.087	(0.031)	
Human Δ1-31	0.068	(0.012)	0.6012
Rat full-length	0.114	(0.010)	
Rat Δ1-29	0.038	(0.026)	0.0546
*FTα with GGT1β*			
Human full-length	0.313	(0.026)	
Human Δ1-31	0.363	(0.099)	0.6467

Data are presented as the mean and SEM. *p*-values are determined by t-test (α = 0.05) between full-length and truncated protein

### Interaction and activity of human FTα with GGT1β

Since FTα is physiologically also necessary for GGTase I activity, we also tested for the interaction and activity of the human truncated FTα with its other physiological partner, GGT1β. Co-elution after co-expression and Ni-NTA purification of His-tagged GGT1β showed an interaction with FTα independent of the presence or absence of the PRR (Additional file 1: Fig. S3). We determined kinetic parameters for GGTase I and could show that there are no significant differences either (GGPP K_M_ full-length vs. truncated 1.13 µM and 1.57 µM, *p* 0.196 and dansyl-GCVLL K_M_ full-length vs. truncated 4.5 µM and 2.36 µM, *p* 0.313; Table 3).

### PRR motif comparison

Since the PRR is conserved but has no influence on known functions of the enzyme, we performed motif comparisons for similarities with already characterised PRR motifs.

Analysing the alignment of ten different mammals with JalView gives the consensus sequence QPEQPPPQPHPPPPQPxQQQ. Analysing this sequence with Blastp only points to predicted FTαs from mammals, underlining its uniqueness.

PRRs can be part of different domains. There are six big groups of proline-rich motif binding modules, namely the SH3-domains, the WW-domains, the EVH1-domains, the GYF-domains, the UEV-domains and profilins [16]. They all interact with specific motifs. There are two potential motifs in the N-terminal part of FTα fitting most likely for binding to Src homology (SH)3-domains or to profilins. SH3-domains have two major consensus sequences namely class I ((R/K)xXPxXP) and class II (XPxXPx(R/K)) (X = non-glycine, hydrophobic residue, x = any natural amino acid) [31], but there are also quite a lot of atypical binding motifs [32]. Profilins are 12–16 kDa proteins expressed in all eukaryotic cells that bind to poly-l-prolines promoting the polymerization of actin filaments. Since there are quite a lot of different additional profilin ligands, important roles in different molecular processes, including signal transduction, are proposed [33].

## Discussion

The α-subunit of the FTase of some eukaryotes, like human and rat, has a PRR, others do not show such a region, like e.g. *S. cerevisiae*. Since PRRs can have an influence on protein–protein interaction and therefore on activity and function [34], our goal was to investigate the influence of a PRR deletion on FTα. We found that i) there is a clustering of the PRR within eukaryotes, especially in mammals, ii) there is no altered binding behaviour to its physiological partners FTβ or GGT1β and no homodimerization, iii) there is no altered activity and inhibitability and iv) the PRR has similarities to known motifs like the SH3-binding domain or the profilin binding domain.

Polyproline stretches can build two different helical conformations, polyproline type I and type II (PP_I_ and PP_II_) [35]. Since PP_II_ is favoured in aqueous solutions, they are common in biological contexts like folded proteins and unfolded polypeptides [36, 37]. We analysed the occurrence of the PRR in a broad range of eukaryotes and found that only mammals do have this region, followed by a quite small sort of linker region (13 aas; reference NP_002018.1). After this very variable linker, the sequence continues highly conserved. Interestingly, a small part of the mammals, the marsupials, have an alanine rich region instead of the PRR. It could be shown, that poly-alanine regions can form structures similar to PP_II_ helices as well [38]. Unfortunately, there is no available crystal structure of this N-terminal part of the protein. The first 54 amino acids of the human FTα are so highly flexible, that they are not crystallisable (PDB 2H6F) [30], so that we are not able to compare the structure to this point. Nevertheless, the poly-alanine region could maybe comprise the same role as the PRR. In contrast to the other members of the eukaryotic domain, the N-terminal region of all the tested mammals (N = 10) is highly conserved, whereas the non-mammals (N = 19) have highly divergent N-termini (Additional file 1: Fig.S1).

To unravel the influence of the PRR on the binding behaviour of FTα to its physiological partners, we performed cell-free as well as in-cell experiments. Our findings prove that truncated human as well as rat FTα dimerize with FTβ and GGT1β, respectively, so that the absence of the PRR does not influence the binding of the subunits with their physiological partners. Since a role for truncated FTα homodimers was assumed [14] we tested for homodimerization as well. Our results reveal, that the PRR of FTα does not seem to have an influence on the dimerization of this protein. This was also shown by Hinz et al. [39] taking the interaction of two human α-subunits as a negative control. According to our previous study, truncated FTα did not form dimers, but we observed the formation of homo-oligomers in tests with enriched and purified FTα similar to the full-length protein. We assume that this might be an artificial oligomerization due to the abundant protein excess [40, 41].

Additionally, to the interaction of PRR deficient FTα with its physiological partners and itself, we tested for the activity of FTase with truncated FTα to determine if there is an altered affinity of the enzyme to its substrates after deletion of the N-terminal part. We could show that the K_M_ values for both substrates for human and rat FTase and human GGTase with truncated and non-truncated α-subunits do not show a significant difference. This is in line with the findings by Andres et al. [13], who used methods including radioactive measurements of overexpressed truncated rat FTα in the cytosolic fraction of HEK 293 cells via a discontinuous enzyme assay. Interestingly, Andres et al*.* deleted larger parts of the N-terminus due to their previous studies [12] and could show an increase in activity when deleting 51 N-terminal amino acids compared to the deletion of 39 amino acids. However, measuring the FTase activity in cytosolic extract is complicated due to high amounts of endogenous FTα. Chen et al. also highlighted the PRR but could only find homologues that are obviously unrelated proteins like the catalytic subunits of rat and human protein phosphatase 2B, mouse retinoblastoma-associated protein pplO5, and a fungal protein-tyrosine kinase [12]. To reveal the influence of the PRR on enzyme activity, we screened the few available existing K_M_ values for FPP as substrate for FTase in online databases. Since there are only five organisms consigned in the databases and due to the diverseness of the values, regardless of the PRR (PRR positive: 0.37–46 μΜ; PRR negative: 0.64–8.1 μM), we can conclude, that the PRR has no influence on the affinity of the substrate to the enzyme and therefore maybe on the structure of the protein binding pocket [42–45]. Additionally to our findings on stable K_M_ values, we also show, that the most prominent FTIs lonafarnib and tipifarnib could still inhibit rat and human FTase. Both inhibitors belong to the class of peptidomimetic inhibitors. These findings suggest that the binding pocket for the peptide substrate is not altered by the deletion of the PRR. Nevertheless, we performed in vitro assays with purified protein. Therefore, physiological interaction partners of FTα could be missing, especially with regard to the PRRs role in cell signalling and trafficking. A direct binding of FTα with Vsp4A, a part of the endosomal sorting complex required for transport was reported but this was independent of the PRR [14]. However, an interactome screening let to eleven FTα interaction candidates [46]. Investigating published data on those candidates concerning their binding to either profilins or SH3-domains points to three of them, Nucleosome assembly protein 1-like 1 (NAP1L1), Arf GTPase-activating proteins (ArfGAP1) and erythrocyte membrane protein band 4.1 like 5 (EPB41L5). NAP1L1 is regulated by profilin [47]. ArfGAP1 is member of the Arf GTPase-activating protein family. Proteins of this family, e.g., ASAP1 are well known to harbour PRRs, SH3 binding motifs and SH3 domains [48, 49]. The third candidate, EPB41L5 is in a pathway axis with ASAP1 [50], so maybe FTα plays a role here as well. Therefore, these proteins are of further interest for PRR-related FTα studies.

To get an idea on the possible roles of the PRR of FΤα, we analysed the proline-rich N-terminal parts of human and rat (human: GGE**P**GQ**P**AQ**PPP**Q**P**H**PPPP**QQQ; rat: GGE**P**GQ**P**EQ**PPPPPPPPP**AQQ**P**). Having a closer look at the sequence, even so called “guest” amino acids (histidine, alanine, glycine, asparagine, and glutamine) [51, 52] are partly present in the mammalian consensus sequence QPEQPPPQPHPPPPQPxQQQ (JalView). They could participate in the helical conformation of polyproline helices.

The two proline-rich motifs in the N-terminal part of FTα fit to binding motifs of Src homology (SH)3-domains or to profilins. SH3-binding domains have two major consensus sequences [31], but also quite a lot of atypical binding motifs [32]. Proteins containing an SH3 domain are often involved in the building of protein complexes as well as in signal transduction, vesicular trafficking and protein–protein-interactions [53]. With proteins of Arf GTPase binding family and EPB41L5, discovered by an interactome study, possible candidates harbouring an SH3-binding domain were identified [48–50]. PRRs fulfil different roles and are often related to the phosphorylation of proteins. It is known, that the prenylation activity of FTase and GGTase can be increased by phosphorylation of FTα [54–57], especially by at S60 / S62 [58]. This phosphorylation seems to be insulin-dependent but SH2 independent. SH3 domains show a higher affinity to tyrosine phosphorylation [59], so maybe this N-terminal region plays a role in the phosphorylation of FTα as well. Profilins bind to poly-l-prolines promoting the polymerization of actin filaments. The broad range of different profilin ligands suppose important roles in different molecular processes, including signal transduction [33, 60]. Here, FTα-interacting NAP1L1 would be an interesting candidate regulated by profilin [46, 47].

## Conclusion

In summary, we show here for the first time that an N-terminal PRR of FTα is highly conserved in mammals with the exception of marsupials. The PRR does not influence the binding behaviour of human and rat FTα to themselves or their natural partners FTβ and GGT1β in vitro*.* We could show that the activity and inhibitability is not influenced by the truncation of the N-terminal region in a cell-free assay. Nevertheless, this region shows common binding motifs for other proteins involved in cell-signalling, trafficking and phosphorylation. These very important findings may lead to better understanding of FTase regulation and function. The function of the PRR should be further investigated due to the relevant but only partly understood role of FTα in eukaryotic FTase and GGTase I.

## Supplementary Information

**Additional file 1.** Figs. S1–S4.

## Data Availability

Not applicable.

## Abbreviations

FTase: Farnesyltransferase
FTα: Farnesyltransferase alpha-subunit
FTβ: Farnesyltransferase beta-subunit
GGTase I: Geranylgeranyltransferase
GGT1β: Geranylgeranyltransferase beta-subunit
PRR: Proline-rich region
