# Supplementary material for: Impact of a conserved N-terminal proline-rich region of the α-subunit of CAAX-prenyltransferases on their enzyme properties
Source: Cell Commun Signal. 2022 Aug 8;20:118. doi: 10.1186/s12964-022-00929-w (PMC9358863; doi:10.1186/s12964-022-00929-w)
Supplement: Supplementary file 2 — Additional file 1. Figs. S1–S4. [file 12964_2022_929_MOESM2_ESM.docx]

**Supplement 1.** Alignment and consensus sequence of eukaryotic FTαs. Set of the N-terminal part of eukaryotic FTαs is depicted. The colors are due to conservation (30%) regarding clustalX. The Consensus and the conservation are given below.


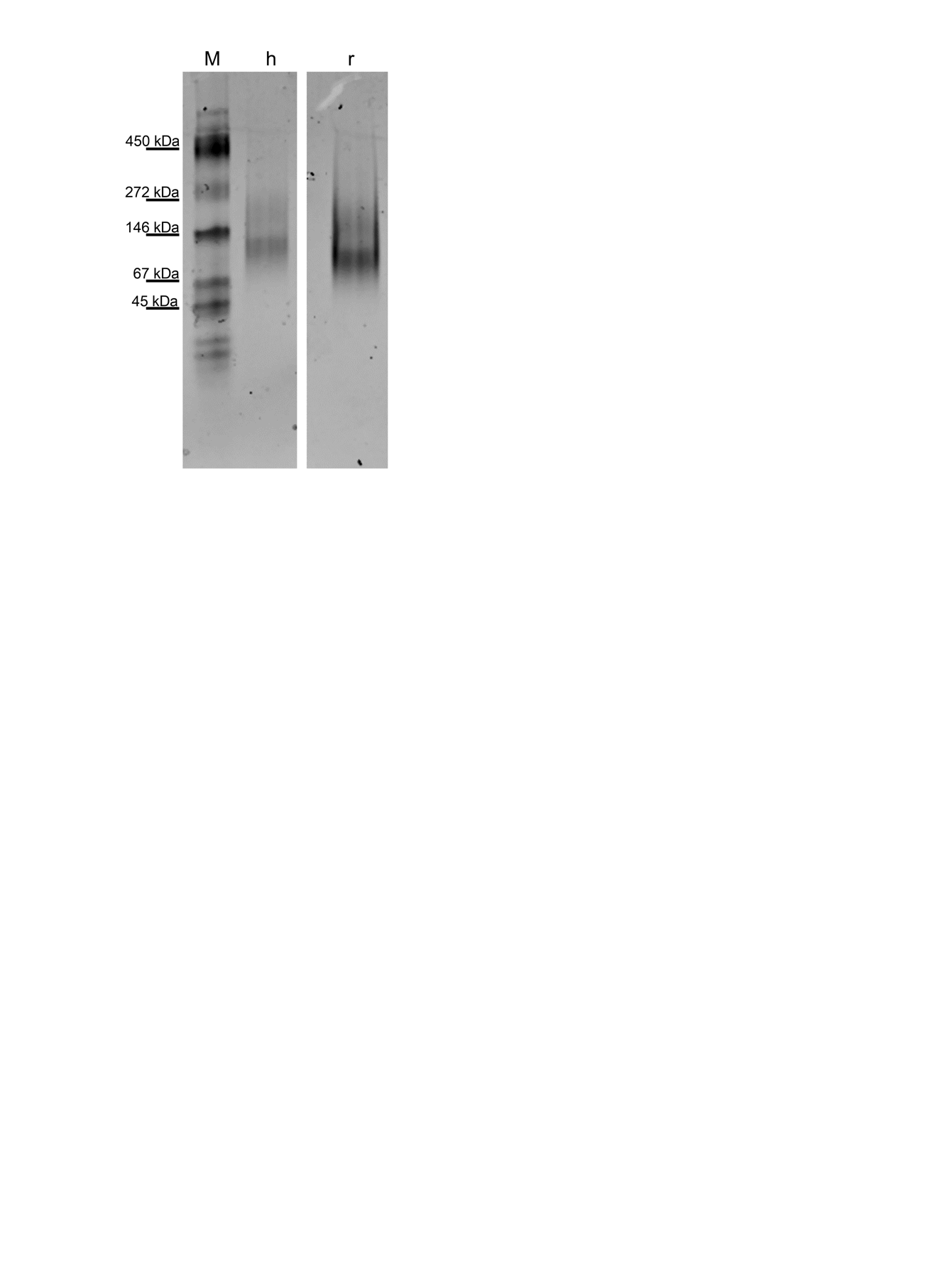


**Supplement 2. Analysis of the purified truncated human and rat FTα**

Native PAGE according to the manufacturer’s protocol (Mini-Protean, Bio Rad, Munich, Germany), Coomassie-stained gel. Human = h, rat = r.


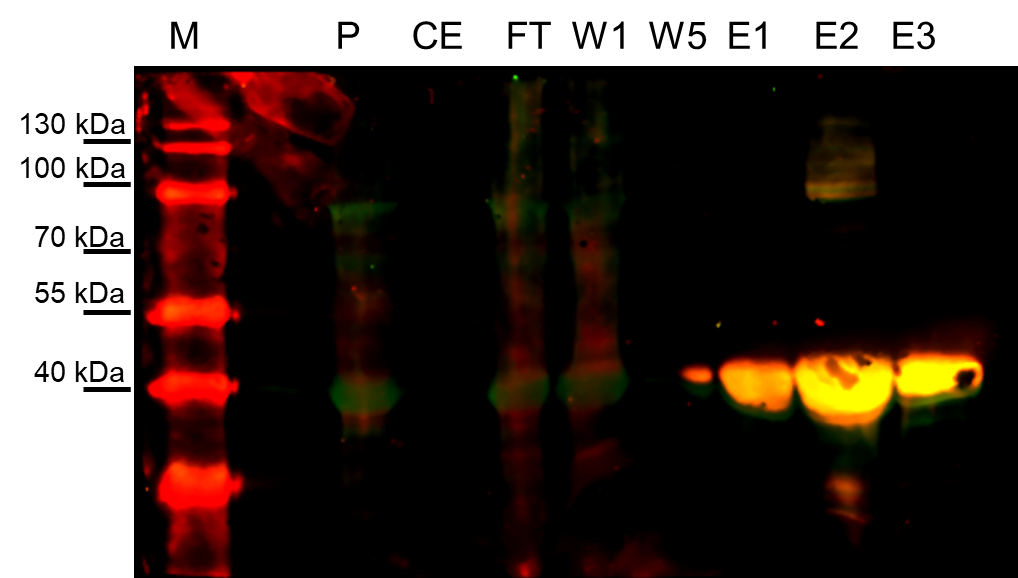


**Supplement 3. Co-purification of truncated FTα with GGT1β**

The samples (pellet (P), crude extract (CE), flowthrough (FT), Wash 1 and 5 (W1, W5) and the elution fractions 1-3 (E1-3)) were applied on an 12% SDS-PAGE blotted on a nitrocellulose membrane. After blocking, the membrane was incubated in 1^st^ anti-FTα antibody [ERP4704, abcam] and afterwards in infra-red second antibody (IRDye® 800CW goat anti-rabbit IgG and a coupled specific His-infra-red antibody (6x-His-tag antibody, 1:2000, DyLight 680, Thermo Fisher Scientific). The blot was visualized by an Odyssey imager (Li-Cor).

CLUSTAL O(1.2.4) multiple sequence alignment

Homo_sapiens -------------------MA--------------ATEGVGEAA--QGGEPGQPAQPP-P 24

Bonobo -------------------MA--------------ATEGVGEAA--QGGEPGQPEQPP-P 24

Dog -------------------MA--------------ATEGVGEAA--QGSESAQPEPPP-P 24

Rat -------------------MA--------------ATEGVGESA--PGGEPGQPEQPPPP 25

Mouse -------------------MA--------------ATEGVGESA--AGGEPGQPEQPPPP 25

Rabbit -------------------MA--------------ATEGVGEAA--AGGEAGQPEQPP-P 24

Cow -------------------MA--------------AADGVGEAA--QGGDPGQPEPPPPP 25

Anteater -------------------MA--------------ATEGVGEAA--QGSEPGQPEQPP-P 24

Dolphin -------------------MA--------------AADGVGEAA--QGGEPGQPEPPPP- 24

Chicken MQY------------PRPRKR--------------RLDGA-MAS--EGMAPAELQAE--- 28

Ostrich -----------------------------------------MAA--EGTAAAPPEPE--- 14

Hummningbird -----------------------------------------MMA--AEGTAV-------- 9

Vombat MVGEATGRGRREGGGESTERGCCRHPDRGEMAAAQVAAEADMVA--EGSAAGEPEQPRPK 58

Koala ------------------------------MAAAQVAAEADMVA--ESSAAGEPEQPRPK 28

Possum ------------------------------MAAAQVVADAEMVT--ESSATGEPERSRRK 28

Shrew_pouch_rat ------------------------------MAAAQVAAEADMVG--ECSAAGEPEPSKPL 28

Komodragon ------------------------------------------------------------ 0

Turtle1 ------------------------------------------------------------ 0

Turte2 ------------------------------------------MV--CGAVPGSVPPLSRL 16

Bat -------------------MA--------------ATEGVGEAT--QGSEPGQPEPPP-P 24

Alligator -----------------------------------------MAA---------------- 3

Bearded_dragon ------------------------------------------MA--DEEGQLDLEPC--- 13

Forest_lizard -----------------------------------------MMA--DGEGGLDPEPC--- 14

Snake -----------------------------------------MMA--DEEERGSPEVC--- 14

Bouchia_clawed_frog -----------------------------------------MLAPGAEEEGGESEVQVAS 19

Caenorhabditis_elegans ------------------------------------------------------------ 0

Microcaecilia_unicolor MAQ------------RRPFVSAFRFPDWE-----GVVSSCGMEA--LEEAG--EEQCRE- 38

Coelacanth -----------------------------------------MSAVEEEK----------- 8

Garfish -----------------------------------------MSG--LEE----------- 6

Sea_lamprey ------------------------------------------------------------ 0

Whale_shark -------------------MAATPAPAES--Q-ADREAQSGALGDGDGEAPGV---DSEP 35

Ant ------------------------------------------------------------ 0

Tigermosquito ------------------------------------------------------------ 0

Salmon_louse ------------------------------------------------------------ 0

Altantic_snow_crab ------------------------------------------------------------ 0

Assel ------------------------------------------------------------ 0

Mulberry ------------------------------------------------------------ 0

Phytoplankton ------------------------------------------------------------ 0

Penicillium ------------------------------------------------------------ 0

Saccharomyces_cerevisiae ------------------------------------------------------------ 0

Cauliflower_fungus ------------------------------------------------------------ 0

Protist ------------------------------------------------------------ 0

Homo_sapiens QP-------HPPPPQQQH-------KEEMAAEAGEAVASPMDDGFVSLDSPSYVLYRDRA 70

Bonobo QPHPPPPQPHPPPPQQQH-------KEEMAAEAGEAVASPMDDGFVSLDSPSYVLYRDRA 77

Dog QPHPPPLQPQ------QQ-------EEAMAAELGEAVASPMDDGFLSLDSPTYVLYRDRA 71

Rat PP--------PPPAQQPQ-------EEEMAAEAGEAAASPMDDGFLSLDSPTYVLYRDRA 70

Mouse PP--------PPPAQQPQ-------EEEMAAEAGEAAASPMDDGFLSLDSPTYVLYRDRA 70

Rabbit QPHPPPAQ------LQQQ-------GEEMAAEAGEAVPSPMDDGFLSLDSPTYVLYRDRA 71

Cow QPHPPPPPPQPPQ-------------------EEAAAASPMDDGFLSLDSPTYVLYRDRA 66

Anteater QPHPPPPQQQ----QLQV-------EEEMAVEAGEAVASPMDDGFLSLDSPTYVLYRDRA 73

Dolphin QPHP--PPPQLPQ-------------------E-EAAASPMDDGFLSLDSPTYVLYRDRA 62

Chicken --------------GGTA-------VE-PE-----SGFGQLLEEEE--EDGGYVLYRDRK 59

Ostrich --------------GG---------GE-PE-----GGFGQLLEEEEEGDDGGYVLYRDRK 45

Hummningbird ------------VIEWQS-------EG-EGGAESEGEFAELLEDKETAKDTGYVLYRDRK 49

Vombat ----P--KTEPAA---AAAAA---AAAAVEAEEVESDSFPEDYGLMRLDSPTYVLYRDRP 106

Koala ----P--KTEAVA---AAAAAAAAAAAAAAAAEAESDSFPEDYGLMRLDSPTYVLYRDRP 79

Possum ----T--E-----------------AAAAELEEVEDDSFPEDYGMMRLDSPTYVLYRDRP 65

Shrew_pouch_rat ----KSPKSEPETVAAVAAAVAAAAAAAAEAEEAEGDSFPEDYGLMRLDSPTYVLYRDRP 84

Komodragon ---------------------------------------------MPYFYWIYIFYRDRK 15

Turtle1 --------------MAEV-------EG-PEARDGEPEPEPELEEGEPLEPGGYLLYRHRK 38

Turte2 ------------AAMAEA-------EG-PEARDGEPQ-EPELEDGEPLEPGSYLLYRHRK 55

Bat QPHTPP--PQ------QQ-------DEVMAAEAGEAVASPMDDGFLSLDSPTYVLYRDRA 69

Alligator ----------------ES-------EGRPPARDGEG-------DGDGDGGGGDSDAEDRE 33

Bearded_dragon ------------ASRLET-------EA-EA-----GGDSELLDCGVPIESNGYTLYRDRK 48

Forest_lizard ------------APHLEA-------EG-EA-----AGDSE-LDSGLPLETDGYVLYRDRK 48

Snake ------------TLRLRT-------EA-DA-----AGDGELLECGEPLDSAGYVLYRNRK 49

Bouchia_clawed_frog QE----------QGEWEF------EQKLPSAPEMDEAVDSDLDYGSLIGSDRYILYRDRK 63

Caenorhabditis_elegans --------------------------------------------MSDSDIPSSTLYKDNV 16

Microcaecilia_unicolor ----------------SG-------ESHEPSKEEEEEEEEEEEEERGSESAAYVLYRDRK 75

Coelacanth ----SP------SVE----------LEPKNENAAEGEVDSEGDYGSAMEIQGYVLYSDRA 48

Garfish ------------IPHLNS------EVQEDGHNEAGGEVEAEDDYVADLCIGGYVFYRNRK 48

Sea_lamprey ----------------------MDAQEAAAAAVAVDDVENSDDEAETRHRAVYVFYRDRV 38

Whale_shark ETEPEPQLQ---GPAWEE----------LKAESGRRASGDSESGAYGRRNDRYIFYRDRK 82

Ant ---------------------------------MSDSSDDELNSNDDHEKTSWILYKDRV 27

Tigermosquito --------------------------------------MADGNSSDEDFPDDWTLYANRP 22

Salmon_louse ------------------------------------------MSSSEDEDSVWIPYSERD 18

Altantic_snow_crab ---------------MGS---TAKTEDTPRQA---PAEDQDDGQDGSSDGEAWVLYRDRK 39

Assel -------------------------------------MSVIESDNSSSAEEEYIFYRDRP 23

Mulberry ------------------------------------------------------------ 0

Phytoplankton --------------------------------------------MVDGDLGAPLLLSKRG 16

Penicillium ---------------------------------------------------MEGTYSTDP 9

Saccharomyces_cerevisiae --------------------------------------------------------MEEY 4

Cauliflower_fungus -----------------------------------------MSTTFDEDEDIETLYADKA 19

Protist ---------------MEADLDVLKECEGVNT----QVTCERLNFPPEPFEVDLDMLNDDK 41

Homo_sapiens EWADIDPVPQNDG----PNPVVQIIYSDKFRDVYDYFRAVLQRDERSERAFKLTRDAIEL 126

Bonobo EWADIDPVPQNDG----PNPVVQIIYSDKFRDVYDYFRAVLQRDERSERAFKLTRDAIEL 133

Dog EWADIDPVPQNDG----PNPVVQIIYSEKFRDVYDYFRAVLQRDERSERAFKLTRDAIEL 127

Rat EWADIDPVPQNDG----PSPVVQIIYSEKFRDVYDYFRAVLQRDERSERAFKLTRDAIEL 126

Mouse EWADIDPVPQNDG----PNPVVQIIYSEKFRDVYDYFRAVLQRDERSERAFKLTRDAIEL 126

Rabbit EWADIDPVPQNDG----PNPVVQIIYSEKFRDVYDYFRAVLQRDERSERAFKLTRDAIEL 127

Cow EWADIDPVPQNDG----PNPVVQIIYSEKFQDVYDYFRAVLQRDERSERAFKLTRDAIEL 122

Anteater EWADIDPVPQNDG----PNPVVQIIYSEKFRDVYDYFRAVLQRDERSERAFKLTRDAIEL 129

Dolphin EWADIDPVPQNDG----PNPVVQIIYSEKFQDVYDYFRAVLQRDERSERAFKLTRDAIEL 118

Chicken EWADIEPVPQNDG----PNPVVQIIYSEKFRDVYDYFRAVLQKDERSERAFKLTADAIEL 115

Ostrich EWADIEPVPQNDG----PNPVVQIIYSEKFRDVYDYFRAVLQRDERSERAFXLTADAIEL 101

Hummningbird EWADIEPVPQNDG----PNPVVQIIYSEKFRDVYDYFRAVLQRDERSERAFKLTADAIEL 105

Vombat EWADIEPVPQNDG----PNPVVQIIYSEKFRDVYDYFRAVLQRDERSERAFKLTGDAIEL 162

Koala EWADIEPVPQNDG----PNPVVQIIYSEKFRDVYDYFRAVLQRDERSERAFKLTGDAIEL 135

Possum EWADIEPVPQNDG----PNPVVQIIYSEKFRDVYDYFRAVLQRDERSERAFKLTGDAIEL 121

Shrew_pouch_rat EWADIEPVPQNDG----PNPVVQIIYSEKFRDVYDYFRAVLQRDERSERAFKLTGDAIEL 140

Komodragon EWADIEPVPQDDG----PHPVVQIIYSEKFKDVYDYFRAVLQRDERSERAFKLTSDAIDL 71

Turtle1 EWADIEPVPQNDG----PNPVVQIIYSEKFRDVYDYFRAVLQRDERSERAFQLTGDAIEL 94

Turte2 EWADIESVPQNDG----PNPVVQIIYSEKFRDVYDYFRAVLQRDERSERAFQLTGDAIEL 111

Bat EWADIDPVPQNDG----PNPVVQIIYSEKFRDVYDYFRAVLQRDERSERAFKLTRDAIEL 125

Alligator EWADIEPVPQNDG----PNPVVQIIYSEKFRDVYDYFRAVLQRDERSERAFNLTADAIEL 89

Bearded_dragon EWADVEPVPQDDG----PHPVVQIIYSEKFKDVYDYFRVVLQRDERSERAFQLTGDAIDL 104

Forest_lizard EWADVEPVPQDDG----PNPVVQIIYSEKFKDVYDYFRAVLQCDERSERAFNLTGDAIDL 104

Snake EWADIDPVPQDDG----PHPVVQIIYSEKFKDVYDYFRAVLQHDERSERAFKLTGDAIGL 105

Bouchia_clawed_frog EWADVKAVPQDDG----PNPVVQIVYSEKFRDVYDYFRAVLQNDERSERAFKLTTDAIEL 119

Caenorhabditis_elegans DWKDITPIYPSKE----EEVAVKIAVTEDFTDAFAYFRAILIKNEKSDRVMALLEDCIRL 72

Microcaecilia_unicolor EWADIDPVPQDDG----PNPVVQIIYSEKFRDVYDYFRAVLQLDEKSERAFKLTGDAIEL 131

Coelacanth EWADVKPVPQDDG----PKPVVQIAYSEKFRDVFDYFRAVFQQDEQSERALKLTTDAIEL 104

Garfish EWSDIEPLPQDDG----PNPVVKIAYSDKFMDVYDYFRAVLKRDERSERAFALTADAIEL 104

Sea_lamprey EWKDLKPVPQEEG----PNAVVQIAYSDKFQDVFDYFRAVLKCDERSERALLLTADAIAL 94

Whale_shark EWADIKPVPQDDG----PNPVVQIAYSENFKDVYDYFRAVLKLDERSERALSLTTDAIEL 138

Ant EWNDVTPIPQDDG----PHPVVSIAYSEKFKDAYDYFRAILKSSEKSERALALTETCIWL 83

Tigermosquito EWSDIEPLKQDDG----ENPVVMIQYSEKFNDVFSYFRAVISKQEKSVRALGLTQDAARL 78

Salmon_louse EWKDVVPIKQSDD----DN-VARINYTDKFRDVYGYIWAIFAKKEISKRALDLTKDAAEL 73

Altantic_snow_crab EWADVTPVPQDDG----PNAVVKIAYTDGYSDVYDYLRAVVKKGELSERALYLTEDAINM 95

Assel DWKDLTPIPQDDG----PHPVVKIAYTDAFQDVYDYFRAIVARGELSERALLLTEDAIQM 79

Mulberry ---------MYN-CHHCVAPGCIIVTIELLRDKYRPWAIRMMQKERTQRIIN--GGLAIL 48

Phytoplankton EWAGVEPLPLFDG-----VPVAAPRLSAEYADMLGLLGAVQASGERSKRVLDLTEALIYV 71

Penicillium EWASVTPIDLDDGSSSGAMPLATIAYPDEYLEATSYLRAVMAANEMSERALNLTRDVISM 69

Saccharomyces_cerevisiae DYSDVKPLPIETD---LQDELCRIMYTEDYKRLMGLARALISLNELSPRALQLTAEIIDV 61

Cauliflower_fungus EWADVVPVQQYEG----VAPLAPIFYSPAYKDATDYFRGVVKTGEKSARVLELTEHIIRM 75

Protist VWEDLELIEKKSN----EPLLFELKLDILEFRAKSFFKVLIKNREFSTRGLYLTTIIIKF 97

* : * : .

Homo_sapiens NAANYTVWHFRRVLLKSLQKD-------LHEEMNYITAIIEEQPKNYQVWHHRRVLVEWL 179

Bonobo NAANYTVWHFRRVLLKSLQKD-------LHEEMNYITAIIEEQPKNYQVWHHRRVLVEWL 186

Dog NAANYTVWHFRRVLLKSLQKD-------LHEEMNYITAIIEEQPKNYQVWHHRRVLVEWL 180

Rat NAANYTVWHFRRVLLRSLQKD-------LQEEMNYITAIIEEQPKNYQVWHHRRVLVEWL 179

Mouse NAANYTVWHFRRVLLRSLQKD-------LQEEMNYITAIIEEQPKNYQVWHHRRVLVEWL 179

Rabbit NAANYTVWHFRRVLLKSLQKD-------LHEEMNYITAIIEEQPKNYQVWHHRRVLVEWL 180

Cow NAANYTVWHFRRVLLKSLQKD-------LHEEMNYISAIIEEQPKNYQVWHHRRVLVEWL 175

Anteater NAANYTVWHFRRVLLKSLQKD-------LHEEMNYITAIIEEQPKNYQVWHHRRVLVEWL 182

Dolphin NAANYTVWHFRRVLLKSLQKD-------LHEEMNYITAIIEEQPKNYQVWHHRRVLVEWL 171

Chicken NAANYTVWHFRRVLLQSLGKD-------LYEELKYITAIIEDQPKNYQVWHHRRVLVEWL 168

Ostrich NAANYTVWHFRRVLLQSLGKD-------LHEELKYITAIIEDQPKNYQVWHHRRVLVEWL 154

Hummningbird NAANYTVWHFRRVLLQSLGKD-------LHEELKYITAIIEDQPKNYQVWHHRRVLVEWL 158

Vombat NAANYTVWHFRRVLLKSLQKD-------LSEEMNYITAIIEEQPKNYQVWHHRRVLVEWL 215

Koala NAANYTVWHFRRVLLKSLQKD-------LSEEMNYITAIIEEQPKNYQVWHHRRVLVEWL 188

Possum NAANYTVWHFRRVLLRSLQKD-------LSEEMNYITAIIEEQPKNYQVWHHRRVLVEWL 174

Shrew_pouch_rat NAANYTVWHFRRVLLKSLQKD-------LSEEMNYITAIIEEQPKNYQVWHHRRVLVEWL 193

Komodragon NAANYTVWHFRRVLLQSLKKD-------LLEELSYITAIIEDQPKNYQVWHHRRVLVEWL 124

Turtle1 NAANYTVWHFRRVLLHSLKKD-------LNEEINYITAIIEDQPKNYQVWHHRRVLVEWL 147

Turte2 NAANYTVWHFRRVLLHSLKKD-------LREEISYITAIIEDQPKNYQVWHHRRVLVEWL 164

Bat NAANYTVWHFRRVLLKSLQKD-------LHEEMNYITAIIEEQPKNYQVWHHRRVLVEWL 178

Alligator NAANYTVWHFRRVLLQSLKKD-------LQEELKYITAIIEDQPKNYQVWHHRRVLVEWL 142

Bearded_dragon NAANYTVWHFRRVLLQSLKKD-------LHEELSYITAIIEDQPKNYQVWHHRRVLVEWL 157

Forest_lizard NAANYTVWHFRRVLLQSLNKD-------LHEELNYITAIIEDQPKNYQVWHHRRVLVEWL 157

Snake NAANYTVWHFRRVLLQSLKKD-------LNEELNYITAIIEDQPKNYQVWHHRRVLVEWL 158

Bouchia_clawed_frog NAANYTVWHYRRVLLTSLQKD-------LREEMNYITAIIEDQPKNYQVWHHRRVLVELL 172

Caenorhabditis_elegans NPANYTVWQYRRVCLTELGWD-------LKKEMRYLSDIIQESPKNYQVWHHRRFIVETI 125

Microcaecilia_unicolor NAANYTVWYFRRVLLQALHKD-------LNEEMNYVTAIIEDQPKNYQVWHHRRVLVEWL 184

Coelacanth NAANYTVWHFRRVLLQSLKKD-------LTEELNYITITIEDQPKNYQVWHHRRMIVEWL 157

Garfish NAANYTVWHYRRVLLQSLKKD-------LYEEMKYITAIIEDQPKNYQVWHHRRMVVEWL 157

Sea_lamprey NSANYTVWHYRRVLLQSLNKD-------LREELAYVQEIIEDQPKNYQVWHHRRLLVDWL 147

Whale_shark NAANYTVWHFRRVVLQSLNKD-------LNEELMYISDIIEDQPKNYQVWYHRQKIVEWL 191

Ant NPANYTVWQYRREILKTLAKN-------LHEEIKYTDRMIKYNSKSYQVWHHRKVIVEWL 136

Tigermosquito NAANYTVWQYRRDILKALNFN-------LYDELDYIEGVIEDNPKNYQVWHHRRVIVEWL 131

Salmon_louse NPSNYTVWHYRRLLLDELGVD-------LNVERDYCRDIMEENPKCYQVWEHRKKLVEKT 126

Altantic_snow_crab NAANYTVWQYRRKILKNLASN-------LEQELNFCREMIEMNPKNYQVWHHRRVIVEWL 148

Assel NAANYTVWQYRRKIIKHINYD-------LKKEIDFCREMIEINPKNYQVWHHRRVIVEWL 132

Mulberry GTGKARLWQFRRAILEALNVD-------LHGELDFTEVVAKGNSKNYQLWHHRRWVAEKL 101

Phytoplankton NSANYTAWDLRWKCLCQLVEDGDT--DAFEREMEFMEDIAASNAKNYQLWNHRRKCALAI 129

Penicillium NPAHYTVWIYRAKILFALEKD-------LNEELSWLNDVSLKYLKNYQIWHHRQVLLSSK 122

Saccharomyces_cerevisiae APAFYTIWNYRFNIVRHMMSESEDTVLYLNKELDWLDEVTLNNPKNYQIWSYRQSLLKLH 121

Cauliflower_fungus NPAHYSAWQYRYHTLIALGSP-------LEDELRLMDKYAIDFLKTYQVWHHRRLLLTAL 128

Protist NSADYTAWYYRNECMKSLDMD-------LREELDFTRKITMESIKAFQPWNHRRNICTLA 150

. * * : : : * * :* * :*:

Homo_sapiens R-DP---SQELEFIADILNQDAKNYHAWQHRQWVIQEFK-----------LWDN--ELQY 222

Bonobo R-DP---SQELEFIADILNQDAKNYHAWQHRQWVIQEFK-----------LWDN--ELQY 229

Dog K-DP---SQELEFIADILNQDAKNYHAWQHRQWVIQEFK-----------LWDN--ELQY 223

Rat K-DP---SQELEFIADILNQDAKNYHAWQHRQWVIQEFR-----------LWDN--ELQY 222

Mouse K-DP---SQELEFIADILSQDAKNYHAWQHRQWVIQEFR-----------LWDN--ELQY 222

Rabbit K-DP---SQELEFIADILNQDAKNYHAWQHRQWVIQEYK-----------LWDN--ELQY 223

Cow R-DP---SQELEFIADILTQDAKNYHAWQHRQWVIQEFK-----------LWDN--ELQY 218

Anteater K-DP---SQELEFIADILNQDAKNYHAWQHRQWVIQEFK-----------LWDN--ELQY 225

Dolphin R-DP---SQELEFIADILNQDAKNYHAWQHRQWVIQEFK-----------LWDN--ELHY 214

Chicken Q-DP---SQELEFIADILNQDAKNYHAWQHRQWVIQEFK-----------LWDD--ELEY 211

Ostrich Q-DP---SQELEFIADILNQDAKNYHAWQHRQWVIQEFK-----------LWDN--ELEY 197

Hummningbird Q-DP---SHELEFIAVILNQDAKNYHAWQHRQWVIQEFK-----------LWDN--ELVY 201

Vombat K-DP---SQELDFIADILNQDAKNYHAWQHRQWVIQEFK-----------LWDN--ELQY 258

Koala K-DP---SQELDFIADILNQDAKNYHAWQHRQWVIQEFK-----------LWDN--ELQY 231

Possum K-DP---SQELDFIADILNQDAKNYHAWQHRQWVIQEFK-----------LWDN--ELQY 217

Shrew_pouch_rat K-DP---SQELDFIADILNQDAKNYHAWQHRQWVIQEFX-----------LWDL--ELQY 236

Komodragon Q-DP---SQELEFIADILNQDAKNYHAWQHRQWVIQEFK-----------LWDD--ELEY 167

Turtle1 K-DP---SQELEFIAEILNQDAKNYHAWQHRQWVIQEFK-----------LWDN--ELEY 190

Turte2 K-DP---SQELEFIAEILNQDAKNYHAWQHRQWVIQEFK-----------LWDN--ELEY 207

Bat K-DP---SQELEFIADILNQDAKNYHAWQHRQWVIQEFK-----------LWDN--ELQY 221

Alligator Q-DP---SQELEFIADILNQDAKNYHAWQHRQWVIQEFK-----------LWDN--ELEY 185

Bearded_dragon K-DP---SQELEFIADILNQDAKNYHAWQHRQWVIQEFK-----------LWDD--ELDY 200

Forest_lizard K-DP---SQELEFIADILNQDAKNYHAWQHRQWVIQEFK-----------LWDD--ELEY 200

Snake K-DP---SQELEFIANILNQDAKNYHAWQHRQWVIQEFK-----------LWDD--ELEY 201

Bouchia_clawed_frog K-DP---SEELEFTAEILSQDAKNYHAWQHRQWVIQEFN-----------LWDN--ELQF 215

Caenorhabditis_elegans G-ES-AVNDELHFCSEVIRDENKNYHAWQHRQWVVRTFK----------VPLEK--ELTF 171

Microcaecilia_unicolor K-DP---SKELEFTADILSQDAKNYHAWQHRQWVIQEFK-----------LWDN--ELQY 227

Coelacanth K-DP---SQELEFTAEILSQDAKNYHAWQHRQWVIQEFK-----------LWDN--ELQY 200

Garfish N-DS---SQELEFIADILNQDAKNYHAWQHRQWVIQEYK-----------LWEN--ELEY 200

Sea_lamprey K-DA---TDELDFTAEILKQDAKNYHAWQHRQWVIQEFN-----------LWDK--ELDF 190

Whale_shark K-DP---SQELGFISGILKQDAKNYHAWQHRQWVIKEFN-----------LWNN--ELAY 234

Ant Q-DP---SEELAFIETVLCKDAKNYHAWQHRQWCIQTFKQVYFFYRIYIYLYDK--ELEY 190

Tigermosquito N-HP---SKELELTESILNMDAKNYHAWQHRQWAIKTYD-----------LFED--ELQY 174

Salmon_louse N-DP---SKELRFTEIILAKDAKNYHAWQHRLWVVCTYK-----------LFDE--EINY 169

Altantic_snow_crab G-DG---TKELRLTEIIFSQDAKNYHAWEHRQWALRTFK-----------LFEG--ELDY 191

Assel G-DP---SKELLFTRIIFNSDAKNYHAWEHRQWVLI--------------FLMN--ELDY 172

Mulberry G--TSATSNELEFTKKILSLDSKHYHAWSHRQWVLQSLG-----------GWED--ELNY 146

Phytoplankton G--PDARETELEFTAAVLEDDAKNYHAWSHRQAIVRAHG-----------LWDE--EDAF 174

Penicillium AHFPTFPPKEADFLMEMFAQDSKNYHVWTYRHWLVRHFK-----------LWDQPRELED 171

Saccharomyces_cerevisiae P-SP-SFKRELPILKLMIDDDSKNYHVWSYRKWCCLFFS-----------DFQH--ELAY 166

Cauliflower_fungus R-STDAAAAELNFIARALGADAKNYHTWSYRQWVLAHFNN-------EARLWAG--ERAW 178

Protist K-SG---FNEIEYVKLEISTSPKNQCAWGYLTWLVRKFG-----------VLDLFKELEF 195

* : . *: .* : *

Homo_sapiens VDQLLKEDVRNNSVWNQRYFVISNTT-------------------------GYNDRAVLE 257

Bonobo VDQLLKEDVRNNSVWNQRYFVISNTT-------------------------GYNDRAVLE 264

Dog VDQLLKEDVRNNSVWNQRYFVISNTT-------------------------GYNDRAILE 258

Rat VDQLLKEDVRNNSVWNQRHFVISNTT-------------------------GYSDRAVLE 257

Mouse VDQLLKEDVRNNSVWNQRHFVISNTT-------------------------GYSDRAVLE 257

Rabbit VDQLLKEDVRNNSVWNQRYFVISNTT-------------------------GYNDRAVLE 258

Cow VDQLLKEDVRNNSVWNQRYFVISNTT-------------------------GYNDRAILE 253

Anteater VDQLLIEDVRNNSVWNQRYFVISNTT-------------------------GYNDSAILE 260

Dolphin VDQLLKEDVRNNSVWNQRYFVISNTM-------------------------GYNDRAVLE 249

Chicken VDQLLREDVRNNSVWNQRYFVIFNTT-------------------------GYDDPAVLD 246

Ostrich VDQLLREDVRNNSVWNQRHFVIFNTT-------------------------GYDDPAVLD 232

Hummningbird VDQLLQEDVRNNSVWNQRYFVIISTT-------------------------GYDDPAVLD 236

Vombat VDQLLKEDVRNNSVWNQRHFVISNTS-------------------------GYNDPAILE 293

Koala VDQLLKEDVRNNSVWNQRHFVISNTS-------------------------GYNDPAILE 266

Possum VDQLLKEDVRNNSVWNQRHFVISNTS-------------------------GYNDPAILE 252

Shrew_pouch_rat VDQLLKVDVRNNSVWNQRHFVISNTS-------------------------GYNDPAILE 271

Komodragon VEQLLKEDVRNNSVWNQRYFVISNTT-------------------------GYDDPAVLD 202

Turtle1 VDQLLKEDVRNNSVWNQRYFVISNTT-------------------------GYNDPAVLE 225

Turte2 VDQLLKEDVRNNSVWNQRYFVISNTT-------------------------GYNDPAVLE 242

Bat VDQLLKEDVRNNSVWNQRYFVISNTT-------------------------GYNDRAILE 256

Alligator VDQLLKEDVRNNSVWNQRHFVISNTT-------------------------GYDDPAILN 220

Bearded_dragon VEQLLKEDVRNNSVWNQRYFVISNTT-------------------------GYDDPAVLE 235

Forest_lizard VEQLLKDDVRNNSVWNQRYFVISNTT-------------------------GYNDPAVLE 235

Snake VEQLLKEDVRNNSAWNQRYFVISNTS-------------------------GYNDPAVLE 236

Bouchia_clawed_frog VDLLLARDLRNNSAWNQRNFVISNTS-------------------------GYSNSSILD 250

Caenorhabditis_elegans ALHMLLLDNRNNSAYNYRYFLMTLYD-------------------------KTEDASQLD 206

Microcaecilia_unicolor VSQLLKEDVRNNSAWNQRYFVIANTT-------------------------GYSNLAILQ 262

Coelacanth VDQLLEEDVRNNSAWNQRHFVVSSTT-------------------------GYNNSTVLE 235

Garfish VEQLLEDDVRNNSAWNQRHFVIAHTT-------------------------GYNDPAIMD 235

Sea_lamprey VERLLDEDLRNNSAWNERHFVVSRTT-------------------------GYSDSATLD 225

Whale_shark VDQLLEDDLRNNSAWNQRYFVISNTT-------------------------GFDDPTVLD 269

Ant VEQLLNDDVRNNSAWNQRYFVISNTT-------------------------KFEQ-EVIN 224

Tigermosquito VDRLISEDMRNNSAWNERFFVLKHT--------------------------GFSA-DVLE 207

Salmon_louse TDRLLKDDIYNNSAWNHRHAVMDLLEN------------------------GEFSGNALQ 205

Altantic_snow_crab VDRLLEEDVRNNSAWNQRHFAITQTT-------------------------GFTA-EVIQ 225

Assel VEELIQEDIRNNSAWNQRYFVISRTT-------------------------GFTE-DVIK 206

Mulberry CDHLLEEDIFNNSAWNQRYFVITRSPILE------------------------GLESMRE 182

Phytoplankton TLELIGRDVYNNSAWNERMVVMEHRSALCGD----------------------PAALRAK 212

Penicillium VEFLLKADVRNNSAWNHRYMLRFGPRDTSAPDAGMVNAGDLSTAPAEKGRLSVVDEDMID 231

Saccharomyces_cerevisiae ASDLIETDIYNNSAWTHRMFYWVNAK-------------------------DVISKVELA 201

Cauliflower_fungus VEELLDKDVRNNSAWHHRFFVVWLAGVR---------A------------GDEDREEVLK 217

Protist VEFLVSGDVYNNSAWNYKNFIFKYFK-------------------------DDFDLDFMV 230

:: * ***.: :

Homo_sapiens REVQYTLEMIKLVPHNESAWNYLKGILQDR--GLSKYPNLL---NQLLDLQPS------- 305

Bonobo REVQYTLEMIKLVPHNESAWNYLKGILQDR--GLSKYPNLL---NQLLDLQPS------- 312

Dog REVQYTLEMIKLVPHNESAWNYLKGILQDR--GLSRYPNLL---SQLLDLQPS------- 306

Rat REVQYTLEMIKLVPHNESAWNYLKGILQDR--GLSRYPNLL---NQLLDLQPS------- 305

Mouse REVQYTLEMIKLVPHNESAWNYLKGILQDR--GLSRYPNLL---NQLLDLQPS------- 305

Rabbit REVQYTLEMIKLVPHNESAWNYLKGILQDR--GLSKYPNLL---NQLLDLQPS------- 306

Cow REVQYTLEMIKLVPHNESAWNYLKGILQDR--GLSKYPNLL---NQLLDLQPS------- 301

Anteater KEVQYTLEMIKLVPHNESAWNYLKGILQDR--GLSKYPNLL---NQLLDLQPS------- 308

Dolphin REVQYTLEMIKLVPHNESAWNYLKGILQDR--GLSKYPNLL---NQLLDLQPS------- 297

Chicken REVQYTLEMITAVPHNESAWNYLKGILQDR--GLSKYPNLL---EQLLSLQPS------- 294

Ostrich REVQYTLEMITAVPHNESAWNYLKGILQDR--GLSKYPNLL---EQLLDLQPS------- 280

Hummningbird REVQYTLDMITAVPHNESAWNYLKGILQDR--GLSKYPNLL---EQLLNLQSS------- 284

Vombat REVQYTLEMIKTAPHNESAWNYLKGILQDR--GLSKYPNLL---EQLLDLQPS------- 341

Koala REVQYTLEMIKTAPHNESAWNYLKGILQDR--GLSKYPNLL---EQLLDLQPS------- 314

Possum REVQYTLEMIRTAPHNESAWNYLKGILQDR--GLSRYPNLL---EQLLDLQPS------- 300

Shrew_pouch_rat REVQYTLEMIKTAPHNESAWNYLKGILQDR--GLSKYPNLL---EQLLDLQPS------- 319

Komodragon REVQFTLEMIKMVPHNESAWNYLKG----------------------------------- 227

Turtle1 REVQYTLEMIKTVPHNESAWNYLKGILQDH--GLSKYQNLL---EQLVDLQPS------- 273

Turte2 REVQYTLEMIKTVPHNESAWNYLKGILQDH--GLSKYQNLL---EQLLDLQPS------- 290

Bat REVQYTLEMIKLVPHNESAWNYLKGILQDR--GLSKYPNLL---NQLLDLQPS------- 304

Alligator REVQYTVEKIKAVPHNESAWNYLKGILQDR--GLSKYPDLL---EQLLDLQPS------- 268

Bearded_dragon REVQYTLEMIRMVPHNESAWNYLKGILQDR--GLSKYPRLL---EQLLELQPS------- 283

Forest_lizard REVQFTLEMIKMVPHNESAWNYLKGILQDR--GLSKHPNLL---GQLLELQPS------- 283

Snake REIQYTLGMIKLVPHNESAWNYLRGILQDR--GLSKYPDLL---EQLLELQPS------- 284

Bouchia_clawed_frog REVQYAIEMIKVAPHNESAWNYLRGILQER--GMSEYPNLL---EQIQRLQQT------- 298

Caenorhabditis_elegans IEINLAKKFIENIPNNESAWNYLAGLLITN--GVTSNSDVV---SFVEDLYET------- 254

Microcaecilia_unicolor REVQYSLEMIKRAPHNESAWNYLRGLLQDQ--GLSQYPNLL---EQILELQHT------- 310

Coelacanth REVQFTLELIRKAPHNESAWNYLKGILQDR--SMSAYPNLL---EKILELYQT------- 283

Garfish REVQYCLQSIRKAPQNESAWNYLKGILQDR--ALSARPGLL---EEILELRKT------- 283

Sea_lamprey REVHYTLGMIKKAPHNESSWNYLRGILEEK--GLARYPSLL---ELIEEIRDGQ------ 274

Whale_shark REVEYTLKMIKQAPHNESAWNYLKGILQDQ--ILSKYPNLL---EQILQLKEQ------- 317

Ant REVDFTLEKIELEKGNESAWNYLRGILSHH--SKGLGLGYN---EKVRHKCEE------- 272

Tigermosquito REINYVMNRIRLIKNNESPWNFLRGLLQQGDGKLGQFPEVV-------DFCED------- 253

Salmon_louse SEVNFALEAIESVVDNESSWSYLTTLLEKS--SSKDYPEIN---DKINKFIQK------- 253

Altantic_snow_crab REVGYTKAAIAKVADNESPWSYLRGVVQHCEGPLGGVGE-------LGAWCQTL------ 272

Assel KEINYTKAAIEKLGNNESSWNYLKGILIHSSSRLSELED-------LEKWCWDL------ 253

Mulberry SEVNYAVRAVIARPENESSWRYLRGLYKDDSGSWVNDSQVS-------SLCLKV------ 229

Phytoplankton EAAGVVSEAVLLAPNNPAPYNYLRGLYARLGPKQPLCADDN------------------A 254

Penicillium GELKFAQEALLRAPENRSPWWYARGVLRAAGRGLGEWEEFV---SGFVSEG--------- 279

Saccharomyces_cerevisiae DELQFIMDKIQLVPQNISPWTYLRGFQELFHDRLQWDSKVV---DFATTFIGD------- 251

Cauliflower_fungus RELAFAKEKIALAPNNLSAWNYFRGVLEHTHTPFALLTAFV---EPYTAAEPPTTTKEQS 274

Protist KELGKDFQRLLKRTDNEGLCSYIIDMVQFLEKSYNKCIMTNCECEGPLSICLVK------ 284

: * . :

Homo_sapiens --------------HSSPYLIAFLVDIYEDMLENQCDNK--------E-----DILNKAL 338

Bonobo --------------HSSPYLIAFLVDIYEDMLENQCDNK--------E-----DILNKAL 345

Dog --------------HSSPYLIAFLVDIYEDMLENQCDNK--------E-----DILNKAL 339

Rat --------------HSSPYLIAFLVDIYEDMLENQCDNK--------E-----DILNKAL 338

Mouse --------------HSSPYLIAFLVDVYEDMLENQCDNK--------E-----DILNKAL 338

Rabbit --------------HSSPYLIAFLVDIYEDMLENQCDNK--------E-----DILNKAL 339

Cow --------------HSSPYLIAFLVDIYEDMLENQCDNK--------E-----DILNKAL 334

Anteater --------------HSSPYLIAFLVDIYEDILENQCDNK--------E-----DILNKAL 341

Dolphin --------------HSSPYLIAFLVDIYEDMLENQCDNK--------E-----DILNKAL 330

Chicken --------------HSSPYLIAFLVDIYEDMLENQCENK--------E-----ETLNKAL 327

Ostrich --------------HSSPYLIAFLVDIYEDMLENQCDNK--------E-----ETLNKAL 313

Hummningbird --------------VSSPYLVAFLVDIYEDMLETQCDNK--------E-----ETLNKAL 317

Vombat --------------HSSPYLIAFLVDIYEDMLENQCDNK--------E-----DTLNKAL 374

Koala --------------HSSPYLIAFLVDIYEDMLENQCDNK--------E-----DTLNKAL 347

Possum --------------HSSPYLIAFLVDIYEDMLENQCDNK--------E-----DTLNKAL 333

Shrew_pouch_rat --------------HSSPYLIAFLVDIYEDMLENQCDNK--------E-----DTLNKAL 352

Komodragon ------------------------------------------------------------ 227

Turtle1 --------------HSSPYLIAFLVDIYEDMLENQCDNK--------E-----ETLNKAL 306

Turte2 --------------HSSPYLIAFLVDIYEDMLENQCEDK--------E-----ETLNKAL 323

Bat --------------HSSPYLIAFLVDIYEDMLENQCDNK--------E-----DILNKAL 337

Alligator --------------HSSPYLIAFLVDIYEDMLENQCSNK--------E-----DILNKAL 301

Bearded_dragon --------------HSSPYLIAFLVDIYEDMLENQCENK--------E-----ETLNKAL 316

Forest_lizard --------------HSSPYLIAFLVDIYEDMLENECENK--------E-----ETLCKAL 316

Snake --------------HNSPYLIAFLVDIYEDMLENQSENK--------E-----ETLSKAL 317

Bouchia_clawed_frog --------------HSSPYLYAFLVDIYEDMLEKKCQNV--------E-----DTLNHAL 331

Caenorhabditis_elegans --------TPEE--KRSPFLLAFIADMMLENIENQKSAE--------ESAGRAKKLYKDL 296

Microcaecilia_unicolor --------------HSSPYLMAFLVDIYEDMLENECDNK--------K-----TTLNQAL 343

Coelacanth --------------HSSPYLLAFLIDLYEDMLENGCNNR--------E-----EILNQAL 316

Garfish --------------HSSPYLFAFLVDLYEDMLESKSSNQ--------E-----EPLTKAL 316

Sea_lamprey -------VSENV--TVSPYLLAFIVDLHEDQLDSGCEDR--------A-----ETLATAL 312

Whale_shark --------------FSSPYLIAFLIDIYEDLLENNCENK--------E-----EIVNKAL 350

Ant -------MYKEG--CRSNHLLACMIDICQE----RCPSD--------ETPSPLFHINFAY 311

Tigermosquito -------LYDSG--IRSPYLLAFLVDLYEEKFFEVKQAG--------ND--PEEYRVKVQ 294

Salmon_louse -------LYDEK--IVNIYMLGAWIDLHEFWIKKDPNNI--------E-----VHLNKIQ 291

Altantic_snow_crab -------HD-DG--QRSSHLLTFMLDLMEDQMERQP-----------DE--RPSLLKRCI 309

Assel -------YKVRG--ETCPFLISFLIDLLEDKMEASTMDE--------NE--RRQNLEQAL 294

Mulberry -------LSTKS--SSRVFALSTLLDLISYGFQPSQEFRDVVNDLVGSPPGPDSNL--AE 278

Phytoplankton IKVAEAALSKD---PAAVPAVELLADFYSDLSMDGAQNT--------DAAQQAAA----- 298

Penicillium -------------AVKSSHAVEWLADVFAEGNGKEA---------------------DAV 305

Saccharomyces_cerevisiae VLSLPIGSPEDLPEIESSYALEFLAYHWGA---DPCTRD------------------NAV 290

Cauliflower_fungus VLDLENPLPSEGADLPSVCALEFLADIHEQAGG--------------------DQTAKAV 314

Protist IIK----L-----QPPSLQLLRLLKKLKP-----------------------DEEVLHQ- 311

Homo_sapiens EL--CEILAKEKDTIRKEYWRYIGRSLQSKHSTENDSPTNVQQ---------- 379

Bonobo EL--CEILAKEKDTIRKEYWRYIGRSLQSKHSTENDSATNVQQ---------- 386

Dog EL--CEILAKEKDTIRKEYWRYIGRSLQSKHSTESDPPTNVQQ---------- 380

Rat EL--CEILAKEKDTIRKEYWRYIGRSLQSKHSRESDIPASV------------ 377

Mouse EL--CEILAKEKDTIRKEYWRYIGRSLQSKHCRESDIPASV------------ 377

Rabbit EL--CEILAKEKDTIRKEYWRYIGRSLQSKHSTESALPTDVQQ---------- 380

Cow EL--CEILAKEKDTIRKEYWRYIGRSLQSKHSTESDPPTNVQQ---------- 375

Anteater EL--CEILAKEKDTIRKEYWRYIGRSLQSKHSTESDPPSKAQQ---------- 382

Dolphin EL--CEILAKEKDTIRKEYWRYIGRSLQSKHSTESDPPTNVQQ---------- 371

Chicken EL--CEILAKEKDTIRKEYWRYIGRSLKNKHSSNTEQSTVTDKQQ-------- 370

Ostrich EL--CEILAKEKDTIRKEYWRYIGRSLKNKHCSGTEQSTPTDKQQ-------- 356

Hummningbird EL--CEILANE-DSIRKEYWRYIGRSLKSKYSSGTEQSTDTQAAVTEHDGEHC 367

Vombat EL--CEILAKEKDTIRKEYWRYIGRSLQNKHSTEGSLP--TDKQQ-------- 415

Koala EL--CEILAKEKDTIRKEYWRYIGRSLQNKHSTEGSLP--TDKQQ-------- 388

Possum EL--CEILAKEKDTIRKEYWRYIGRSLQNKHSTEGSLP--TDKQQ-------- 374

Shrew_pouch_rat ESEVCRPQRRIYTTL-------------------------------------- 367

Komodragon ----------------------------------------------------- 227

Turtle1 EL--CDILANEKDTIRKEYWKYIGRSLQNKHSTGSEPDLPTDPQQ-------- 349

Turte2 EL--CNILAKEKDTIRKEYWKYIGRSLQNKYSTGTEQDLRTDQQQ-------- 366

Bat EL--CEILAKEKDTIRKEYWRYIGRSLQSKHSTESDPPTNVQQ---------- 378

Alligator EL--CEILAKEKDIIRKEYWRYIARSLQNKHSTCTEQSLPVDAQQ-------- 344

Bearded_dragon EL--CEILAKEKDVIRKEYWRYMGRSLKSKHSPNEEQNIQSESDQQ------- 360

Forest_lizard EL--CEILAKEKDIIRKEYWRYIGRSLKSKHSPSEEQNVPTYNDQE------- 360

Snake EL--CEILSKEKDIIRKEYWGYIGRSLKSKYSPNEEQTVPTENDQH------- 361

Bouchia_clawed_frog EL--CDILAKEKDTIRKEYWRYIGRSLTVKYGVNRTEKEEPIHVDIVQPE--- 379

Caenorhabditis_elegans QS---------IDPVRVNYYRHQSLLAQTML--IKAQTKVTAK---------- 328

Microcaecilia_unicolor EL--CEMLAKERDTIRKEYWRYIGRCLQKKYSASSEDGETSPSSSTKQQQ-HF 393

Coelacanth EL--CELLANEKDTIRKEYWRYIGRSLKSKCSIGVESSDVTDGPSVVEQ---- 363

Garfish EL--CELLAEEKDSIRKEYWRYIGRSLKDKYGTSEESTTPLQEHQENCGND-- 365

Sea_lamprey KL--CEQLATELDMIRGEYWRYVANSLQQRFGPPAASTKEPSARPVQATA--- 360

Whale_shark EL--CECLAVKKDTIRQEYWRYFARCLKEKYGVKQEDTEPTESEASLQEGCSN 401

Ant EL--CKDLSKKYDTIRWRYWDYVASQLAIKLKTESSD---------------- 346

Tigermosquito DL--CETMATQHDKIRCKYWRYIAENFRRKIDAEENQNGL------------- 332

Salmon_louse EV--VELLAKKFDVGREKYWTYLGKKISNDYRNCAVTSSTT------------ 330

Altantic_snow_crab EM--CESLATEHDKIRREYWRYIERNLSHRFGA-------------------- 340

Assel KL--CESLAGEHDKIRREYWRFIAKRLLSKFKM-------------------- 325

Mulberry TV--C-ALLEREDPTRANYWRWRRSKLP------------------------- 303

Phytoplankton ----LFSMLKTSDPIRARYWQHRQAELVLE----------------------- 324

Penicillium RM--LTMLKEEFDPIRKNYWDYRIRKLDQAVA--------------------- 335

Saccharomyces_cerevisiae KA--YSLLAIKYDPIRKNLWHHKINNLN------------------------- 316

Cauliflower_fungus EI--WKTLANKHDTMRKKYWEYRIREALQAKAS-------------------- 345

Protist --------MAILDPIRSDFYKISKSLP-------------------------- 330

**Supplement 4. Alignment of a representative set of FTα sequences.**

Alignment was performed by the online tool ClustalO
